# Trauma-Informed Care for Intimate Partner Violence and Sexual Assault: Simulated Participant Cases for Emergency Medicine Learners

**DOI:** 10.15766/mep_2374-8265.11500

**Published:** 2025-02-25

**Authors:** Bridget Matsas, Alysa Edwards, Eleanor M. Birch, Stefani Ramsey, Hailey Benesch, Shane Goller, Jillian Phelps

**Affiliations:** 1 Third-Year Resident, Department of Emergency Medicine, Madigan Army Medical Center; 2 Second-Year Resident, Department of Emergency Medicine, Madigan Army Medical Center; 3 Nurse Educator and Curriculum Coordinator, Anderson Simulation Center and Army Central Simulation Committee, Madigan Army Medical Center; 4 Assistant Director of Quality Improvement, Department of Emergency Medicine, Madigan Army Medical Center; 5 Program Director, Department of Emergency Medicine, Madigan Army Medical Center

**Keywords:** Sexual Assault, Simulated Participant Encounter, Trauma-Informed Care, Emergency Medicine, Intimate Partner Violence, Quality Improvement/Patient Safety, Simulation

## Abstract

**Introduction:**

Emergency medicine (EM) providers often care for patients who present with concerns related to sexual assault (SA) or intimate partner violence (IPV). However, many providers feel uncomfortable discussing SA and IPV with patients. We aimed to design a curriculum using trauma-informed care principles to improve self-assessed competency in caring for this patient population.

**Methods:**

EM learners, including residents, EM physician assistant fellows, and medical students, attended a 25-minute didactic session introducing the concept of trauma-informed care and important questions to ask in IPV and SA cases. Learners then participated in a 15-minute simulated single-patient encounter during which they practiced collecting a trauma-informed history identifying features of IPV or SA and appropriately responding to such disclosures. The encounters were observed by a trained SA medical forensic examiner or a victim advocate. The learners next participated in a 10-minute individual and 15-minute group debrief.

**Results:**

Sixteen pre- and 17 postcurriculum self-assessments were completed. There was a statistically significant increase in self-perceived confidence in the learners’ ability to collect information (*p* < .01), use strategies to help patients feel physically and psychologically safe (*p* < .001), recognize how bias influences patient encounters (*p* < .05), and provide counseling (*p* < .05). Learners overall found the learning exercise valuable.

**Discussion:**

The exercise introduced learners to trauma-informed care, improved learner confidence, and was well received. Many EM residency programs incorporate simulation into their curriculum; this simulation exercise can be adapted to other programs’ educational needs.

## Educational Objectives

By the end of this activity, learners will be able to:

1. Recognize the role of emergency department providers in responding to disclosures of intimate partner violence (IPV) and sexual assault (SA).
2. Apply trauma-informed history-taking skills during a simulated patient encounter.
3. Increase confidence in taking a history from patients presenting with concerns related to SA and IPV.

## Introduction

Sexual assault (SA) and intimate partner violence (IPV) are prevalent public health issues with significant negative health effects. Patients may present with physical injury, post-traumatic stress disorder, sexually transmitted infections, or other chronic health problems.^1,2^ According to the Centers for Disease Control and Prevention, 47.3% of women and 44.2% of men have experienced IPV, and up to 26.8% of women and 3.8% of men report a completed or attempted SA in their lifetime.^1,2^ IPV is defined as “any physical or sexual violence, stalking, and/or psychological aggression by a current or former dating partner or spouse.”^2^ SA is defined as “a sexual act that is committed or attempted by another person without freely given consent of the victim or against someone who is unable to consent or refuse.”^3^ There is significant overlap between the two forms of violence; according to the 2016/2017 National Intimate Partner and Sexual Violence Survey, 39.3% of rapes reported by women and 12.5% reported by men were perpetrated by intimate partners.^1^

While SA- and IPV-related emergency department (ED) encounters are increasing, seeking care in the ED can be retraumatizing for patients.^4,5^ Trauma-informed care (TIC), a framework described by the Substance Abuse and Mental Health Services Administration (SAMHSA), seeks to minimize the impact of trauma at all levels of patient care.^6^ The framework outlines six principles: (1) safety; (2) trustworthiness and transparency; (3) peer support; (4) collaboration and mutuality; (5) empowerment, voice, and choice; and (6) cultural, historical, and gender issues.^6^

Current literature suggests that few ED providers feel adequately trained in TIC or comfortable discussing IPV or SA.^7,8^ Simulation-based interventions may enhance providers’ abilities by encouraging the practice of critical skills in a controlled environment.^9^ Despite increasing recognition of the importance of TIC in the ED, only one simulation exercise for residents was identified in a 2022 systematic review of TIC use in emergency medicine (EM).^10^ *MedEdPORTAL* has published interventions focused on TIC in encounters related to SA or IPV, but there are limited trainings in the acute care setting.^11–15^ Our primary aim was to create a simulation curriculum to enhance EM learner self-assessed competency and comfort in caring for patients who have experienced IPV or SA.

## Methods

Our institution's Human Research Protections Office approved this project as a quality improvement initiative with a “Not Research” designation exempt from further review (Protocol# 223001, October 3, 2022). The curriculum was developed by residents, faculty, and the simulation team within the Madigan Army Medical Center Department of Emergency Medicine. The exercise was integrated into the department's monthly simulation training conducted at the Anderson Simulation Center on Joint Base Lewis-McChord, WA. The center was accredited by the Society for Simulation in Healthcare and adhered to the Standards of Best Practice established by the Association of Standardized Patient Educators (ASPE).^16^ Learners were EM residents, rotating medical students, and EM physician assistant fellows. Learners were expected to have a general knowledge of medical history-gathering before participating.

### Development

We developed a 25-minute didactic session (Appendix A) to provide key definitions and statistics related to SA and IPV, introduce the TIC framework, and review history-gathering techniques. We used SAMHSA's trauma-informed approach because it was widely accepted, provided a clear definition of trauma, and outlined a comprehensive framework for addressing the impact of trauma.^6^ This exercise was conducted for learners who primarily worked at a military treatment facility; the didactic session included military definitions of SA and IPV and reviewed documentation specific to military treatment facilities. Didactic content focused on collecting a trauma-informed history and interacting with patients rather than definitive patient disposition or case management, which were determined to be outside the scope of this initiative.

We conducted a literature review of existing scenarios that related to IPV, SA, and TIC and created patient encounters specific to the needs of our learners.^14,17^ While patients experiencing IPV and SA can present to the ED with varying physical and psychological concerns, this curriculum focused on physical injuries as they provided a clear, acute context for applying TIC principles and established foundational skills to recognize underlying patterns of abuse.

We used four scenarios (Appendix B). Cases 1 and 2 involved a patient presenting with abdominal pain or wrist pain, respectively. In both scenarios, the patient was initially hesitant or uncomfortable in describing the events leading up to their injuries, challenging the learner to recognize signs of IPV and employ TIC strategies. Cases 3 and 4 involved more explicit concerns regarding SA while still providing an opportunity to practice applying TIC principles and collecting a patient history. All scenarios incorporated active-duty service members or their dependents to reflect the learners’ primary patient population. The military status of the patients added social and professional complexity to the cases, encouraging learners to consider issues like the physical safety of a patient returning to the barracks and/or the patient's role in the reporting process. The scenarios could be generalized to civilian patient populations by removing the military status and focusing solely on the presenting injury. This adaptation would still permit nonmilitary learners to practice caring for patients experiencing IPV or SA using TIC principles. Further case adaptations could be made by altering the patients’ gender or sexual orientation.

### Personnel

We worked with a nurse educator from the Anderson Simulation Center to develop and implement this curriculum. The nurse educator was a member of the ASPE and adhered to the ASPE Standards of Best Practice to train and prepare the simulated participants (SPs).

Each scenario involved one SP per room (four total). The SPs were recruited from a company contracted to provide SPs for medical simulations; the subject matter was disclosed so that SPs could make an informed decision regarding their participation. One week prior to the encounter, the nurse educator met with the SPs to review the scripts and conduct rehearsals. SPs were instructed to pay attention to learner behavior and to specifically respond if the learner was perceived as dismissive or insensitive. The SPs were allowed to opt out at any time. Following the event, the SPs were invited to participate in a debrief of their role; the nurse educator provided contact information for resources and follow-up if the SPs needed additional support.

Two timekeepers were present to ensure the simulation encounters and feedback sessions started and ended on time. An observer with advanced training in interviewing patients with concerns related to SA or IPV, either an SA medical forensic examiner (SAMFE) or a victim advocate (VA), was stationed in each room in order to assess both verbal and nonverbal communication between the SP and learner that might not have been observable via monitoring equipment. These observers were also present to ensure the safety of both the learner and the SP.

The group debrief was facilitated by three EM faculty physicians and the nurse educator. All faculty facilitators had clinical experience working with patients experiencing IPV or SA and regularly led medical training events with this group of learners. One simulation operation specialist was present and responsible for the operation of audio and video equipment.

### Environment and Equipment

The rooms were set up to resemble standard rooms in the ED. Each room contained the following additional equipment:

• Three seats—one seat for the SP, learner, and observer, respectively.
• Pen and paper.
• Facial tissue.
• Audiovisual capabilities for faculty to observe the encounter in a separate observation room.

### Implementation

Four learners from each group were randomly assigned to each of the four cases. If there were fewer than four learners, one case was not simulated at random. If there were more than four learners, the remaining learners observed the case from the simulation center control room. Each learner participated in a single 15-minute case followed by a 25-minute debrief. There were five iterations, with each SP portraying their respective patient up to five times. Each case was separated by a 5-minute break to allow learners to transition to other simulation events; a longer 30-minute break occurred between iterations three and four.

Prior to the start of the encounter, learners were informed of the sensitive nature of the training to ensure a physically and psychologically safe learning environment. All participants, including SPs and observers, were encouraged to call a time-out at any time to stop the simulation exercise, remove themselves from the scenario, and/or receive further support if needed.

The nurse educator escorted the learners to the exam rooms and briefed them on the patient's chief concern. Learners were informed that while they might perform a physical exam, it was not expected, as the purpose of the exercise was to practice collecting a history. They were instructed not to perform any sensitive exams, including breast, rectal, or genitourinary exams. The learners were briefed that the observer was not a part of the encounter but instead was there to provide feedback.

The observer accompanied the learner into the room where the SP was waiting. The facilitators observed from a separate room with audiovisual capabilities. One facilitator was assigned to each exam room. The timekeepers were present and circulated between the rooms to prompt learners to start and end their patient encounter.

Following the case, learners participated in a 10-minute individual feedback session with the SP and observer. This curriculum was intended to introduce TIC principles and to increase self-assessed confidence caring for patients experiencing IPV or SA; learners were not assessed on achievement of critical actions and instead received formative feedback on their interactions with the SP. While the SPs and observers were not required to use the critical action checklists outlined in Appendix C, it served as a foundation from which to provide such feedback and could be used in future iterations to measure learner performance. There were no postencounter activities such as placing orders or writing notes.

A 15-minute group debrief involving all learners, observers, and faculty facilitators was conducted immediately after the individual feedback sessions. This debrief assessed learners’ overall experience with the simulation rather than achievement of critical actions. The plus-delta format was used to ask the following questions^18^:

• What went well?
• What did not go well and could have been done differently?
• How did the participants feel the overall simulation exercise went?

### Learner Self-Assessment

Learners completed anonymous 13-item pre- and 17-item postcurriculum self-assessment tools (Appendix D) to examine perceived knowledge and comfort in caring for patients experiencing IPV and SA. These assessments were created by adapting questions from the Physician Readiness to Manage Intimate Partner Violence Survey (PREMIS). The PREMIS, a 67-item survey querying physician knowledge, perceptions, and practice issues surrounding IPV, had been shown to be predictive of IPV-related clinical behavior, was reliable across varying physician populations, and was freely available for use by educators.^19^ Items IIa-IIc, were adapted directly from the PREMIS to fit our learning objectives while items IId-IIf were created considering the six guiding principles of TIC.^6^ Item III asked these questions in the context of SA. The postcurriculum self-assessment included additional questions in items IV and V meant to examine learner perception of the use of simulation to teach about IPV and SA. Responses were collected using a 5-point Likert scale (1 = *strongly disagree,* 5 = *strongly agree*). The precurriculum self-assessment tool was delivered immediately prior to the didactic session. The postcurriculum tool was delivered after the simulation had been completed.

Responses were analyzed using R (R Foundation). The Mann-Whitney test compared pre- and postcurriculum self-assessment responses for all respondents while the Kruskal-Wallis test assessed differences in pre- and postcurriculum self-assessment responses by year of training.

## Results

There were 16 pre- and 17 postcurriculum self-assessments completed, contributing to a total of 33 learner responses. Of the assessments collected, 14 (42%) were completed by first-year residents, six (18%) by second-year residents, five (15%) by third-year residents, four (12%) by medical students, and four (12%) by EM physician assistant fellows.

Pre- and postcurriculum self-assessment responses on IPV and SA are displayed in Tables 1 and 2, respectively. We calculated *p* values with the Mann-Whitney test. The Kruskal-Wallis test showed no significant association between respondents’ level of training and assessment tool responses. The postcurriculum Likert-scale scores were significantly higher for most questions. Although the degree of agreement increased, there was no significant difference in response regarding learners’ comfort discussing IPV or SA with patients. Learners’ perceived ability to appropriately respond to disclosures of IPV was significant but not to disclosures of SA.

**Table 1.**
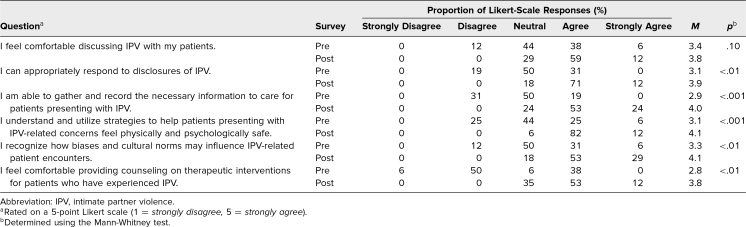
IPV Survey Responses (Presurvey *N* = 16, Postsurvey *N* = 17)

**Table 2.**
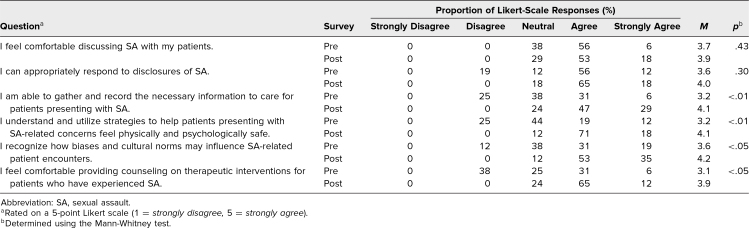
SA Survey Responses (Presurvey *N* = 16, Postsurvey *N* = 17)

The averaged Likert-scale responses regarding the effectiveness of the simulation are illustrated in Table 3. Overall, learners felt that the curriculum was an effective way to improve clinical skills and found the cases realistic.

**Table 3.**
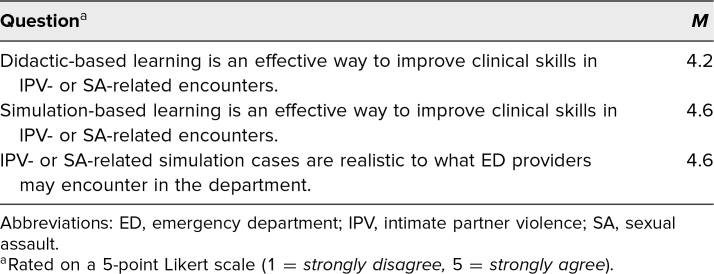
Average Perceived Effectiveness of the Didactic and Simulation-Based Learning Exercises (*N* = 17)

At the end of the self-assessment tool, learners were asked to comment on the training. Learners felt that the SP methodology was valuable, with two respondents requesting additional training with SPs. One learner commented that they appreciated the individualized feedback provided by SPs and SAMFEs. Multiple learners suggested having institution-specific information printed and available during the case to help guide the encounter.

## Discussion

In this quality improvement project, we created a simulation-based curriculum that introduced EM learners to the principles of TIC and provided history-gathering techniques for use with patients experiencing IPV or SA. Analysis of pre- and postcurriculum assessments demonstrated overall improvement in self-assessed ability to care for this patient population. While learners indicated increased comfort in discussing IPV and SA, there was not a statistically significant change. A systematic review by Kalra and colleagues in 2015 observed similar findings—while learners may enhance their abilities to care for patients, self-perceived readiness to respond often remains unchanged.^20^ This may reflect societal attitudes surrounding IPV and SA rather than an inability to provide effective care. Additionally, we found no significant difference pre- and postcurriculum by year of training, which suggests that all learner groups can benefit from simulation-based training on managing cases related to IPV and SA.

Learners reported that simulation-based learning was effective. These findings are consistent with existing literature, which reports that educational interventions involving TIC, IPV, and SA are well received by learners and that the use of simulation improves comfort in screening for IPV, managing patients, and responding to disclosures.^7,12,13,15,21,22^ Other studies have similarly demonstrated that simulation encounters are effective at improving learner comfort when managing cases of SA.^11,23,24^ While this area of research is expanding, there are few simulation-based interventions that focus on teaching EM learners how to effectively care for patients who present to the ED with concerns related to IPV or SA.^7,15,23,24^

The didactic session was important for introducing learners to the TIC framework and interviewing techniques. It also provided an opportunity to emphasize the importance of personal safety and established ways in which learners could step away if negatively impacted by the content.

While this population of learners frequently participates in simulation exercises, manikins or artificial models are often used in lieu of SP methodologies. The success of this SP methodology emphasizes the ability of simulation to create spaces where learners feel empowered to engage in difficult conversations without the risk of retraumatizing patients in a real patient encounter. As a result of learner feedback, SPs will be involved in more simulation exercises in the future.

Considering the complexity and depth of this topic, early involvement of subject matter experts in the planning and development process is recommended. Simulation center staff provided key insight into logistics, resources, and best practices in medical simulation. SAMFEs and VAs offered a thorough understanding of the required administrative tasks, possible emotional and logistical barriers to care, appropriate counseling techniques, and available local resources. Their involvement added fidelity to the simulation and served to introduce learners to invaluable patient team members not always physically present in the ED.

This curriculum was designed for learners working in a military context. At time of publication, it represents the first educational intervention combining simulation training with a TIC framework to prepare EM learners to care for active-duty service members and their families experiencing IPV or SA. Nevertheless, the content was designed to be applicable to both military and civilian patients as all the EM learners who participated work in both military and civilian health care settings. While some scenario details are specific to active-duty patients, these details can be adapted to best reflect an institution's primary patient population.

There are several limitations to this project. The statistical strength of the data was limited by a small sample size and unequal numbers of pre- and postcurriculum responses. To allow adherence to work-hour restrictions, learners arrived and departed from the session at different times such that not all of them attended both the didactic session and the simulation exercise; some learners did not complete either or both the pre- and postcurriculum self-assessments. Additionally, a larger proportion of first-year residents completed the assessments compared to learners of other training levels. This may have impacted the ability to identify a significant difference in responses by year of training. The assessment tool did not track which case the respondent participated in, which limited comparison between cases.

This curriculum aligns primarily with level 1 of the Kirkpatrick training evaluation model as only self-assessed data were collected from a single point in time.^25^ While self-assessed data are frequently collected to measure outcomes of IPV and SA response education, this may not reflect actual proficiency. Finally, this type of data does not assess the impact on patient outcomes or knowledge retention over time. The simulation's effects on knowledge acquisition and retention are areas of potential future study.

This curriculum will be incorporated as a recurring exercise for EM learners. Future iterations will expand on the institution-specific requirements and resources available for patients and incorporate nonphysical injuries into simulation cases. Additionally, we plan to have observers measure learner performance during the simulation as well as knowledge and skill retention over time.

Teaching EM learners about trauma-informed history-gathering techniques using a simulation-based curriculum improved learner self-assessed ability to care for patients experiencing IPV and SA. With the increasing implementation of simulation-based curricula in EM, this content can be adapted and utilized by other medical training programs. We are hopeful that education on TIC will improve the experiences of patients affected by IPV or SA and lower the risk of retraumatization in the ED.

## Appendices


Didactic Lecture.pptxSP Case Development Tool.docxCritical Actions Checklist.docxPre- and Postcurriculum Self-Assessments.docx
*All appendices are peer reviewed as integral parts of the Original Publication.*
