# Supplementary material for: Trauma-Informed Care for Intimate Partner Violence and Sexual Assault: Simulated Participant Cases for Emergency Medicine Learners
Source: MedEdPORTAL. 2025 Feb 25;21:11500. doi: 10.15766/mep_2374-8265.11500 (PMC11850505; doi:10.15766/mep_2374-8265.11500)
Supplement: Supplementary file 1 — Didactic Lecture.pptxSP Case Development Tool.docxCritical Actions Checklist.docxPre- and Postcurriculum Self-Assessments.docx [file mep_2374-8265.11500-s001.zip › B. SP Case Development Tool.docx]

Appendix B: *MedEdPORTAL* Standardized Patient Case Development Tool

User Guide

Timing: 15-minutes per case

Use: Simulation curriculum intended to enhance emergency medicine (EM) learner self-assessed competency and knowledge in caring for patients who have experienced intimate partner violence (IPV) or sexual assault (SA)

Content: Four simulated scenarios involving patients with military affiliation experiencing either IPV or SA

1. An Active-Duty Soldier with Abdominal Pain
2. A Patient with Wrist Pain
3. A Soldier Presents with Rectal Pain
4. A Soldier Presents after a Sexual Assault

Equipment: Examination room with three chairs, pen and paper, facial tissue, audiovisual (AV) capabilities, observation room

Personnel:

- Standardized Participant (SP): recommend one per case (four total) if multiple cases are performed simultaneously; all SPs should be notified of content involving IPV/SA during recruitment process
- Nurse educator: recruit and train SPs, conduct rehearsals, participate in simulation debrief
- Timekeeper: adhere to case/debrief start and end times
- Observer(s): faculty providers, sexual assault medical forensic examiners (SAMFE) and/or a victim advocate (VA) available to assess learner performance during case, ensure safety of learners and SPs; recommend at least one in-person observer per case
- Learners: recommend one learner per case, should be familiar with collecting medical history
- Simulation operations specialist: operates AV and simulation equipment

Potential modifications:

- May be used for non-military contexts by removing patient military affiliation; this may remove some of the specific social complexity associated with IPV/SA in the context of the military, learners will still be able practice interacting and caring for patients experiencing IPV/SA
- Patient gender and/or sexual orientation may be adjusted based on SP identity and/or instructor/learner goals

Appendix B: *MedEdPORTAL* Standardized Patient Case Development Tool Case 1

Date: October 5^th^, 2022

Primary Case Author: Stefani Ramsey

This case was inspired by the objective structured clinical encounter (OSCE) included in Schrier and colleagues’ 2017 MedEdPORTAL publication.^16^ Case entitled “IPV and Abdominal Pain” by Steven Rougas and Sadie Elisseou, retrieved from: https://www.mededportal.org/doi/full/10.15766/mep_2374-8265.10622 on September 14, 2022. Creative Commons License associated: https://creativecommons.org/licenses/by-nc-sa/4.0/legalcode.

Standardized Patient Educator: Stefani Ramsey

Name of Case: An Active-Duty Soldier with Abdominal Pain

Name of Educational Activity: Taking a Trauma-Informed History in Cases Related to Intimate Partner Violence

Patient Name: Sam Jones

Chief Complaint: Abdominal Pain

Most Likely Diagnosis and Differential with Rationale from History and/or Physical Exam:

1. Abdominal Wall Musculoskeletal Hematoma – Suggested by mechanism of injury, location of pain, hemodynamically stable but uncomfortable patient with abdominal wall ecchymosis.
2. Duodenal Hematoma – Suggested by mechanism of injury, location of pain, exam with abdominal wall ecchymosis.
3. Liver Laceration – Suggested by mechanism of injury and location of pain.
4. Splenic Laceration -- Suggested by mechanism of injury and location of pain.
5. Traumatic Pancreatitis – Suggested by mechanism of injury and location of pain.

Domains: Check all that apply

✓ Professionalism

✓ Communication and Interpersonal Skills

✓ Medical History

- Physical Exam

✓ Shared Decision-Making

✓ Patient Education

- Clinical Reasoning
- Documentation
- Handoff
- Presentation

✓ Other: Trauma-Informed Care

Type and Level of Learner: Emergency medicine (EM) learners (medical students, residents, EM physician assistant fellows)

Case Objectives: Please list specific objectives for each of the domains you have checked above:

1. Identify concern for IPV.

2. Respond appropriately to the disclosure of IPV.

3. Establish a psychologically and physically safe environment for the patient using TIC principles.

4. Elucidate if the patient is willing to discuss next steps in managing IPV from the Emergency Department.

5. Offer that the patient may meet with the Victim Advocate.

6. Counsel the patient on resources available to them (if standardized patient is agreeable to further counseling).

| SETTING: | Emergency department examination room |
| --- | --- |
| PATIENT PROFILE: | |
| Age range | 18 - 50 |
| Religious/spiritual background | All may be used |
| Sex (e.g., male, female, intersex, transwoman, transman) | All may be used |
| Sexual orientation (e.g., heterosexual, lesbian, gay, bisexual, pansexual, queer, asexual) | All may be used |
| Gender expression (e.g., man, woman, genderqueer) | All may be used |
| Race and ethnicity | All may be used |
| Physical description (e.g., BMI, height range) | All may be used |
| Physical limitations | None |
| Patient appearance (e.g., disheveled, hospital gown, business casual, casual) | Casual civilian clothes |
| Moulage + location (e.g., none, bruises, scars, body piercing, tattoos) | Ecchymosis over superior aspect of abdomen |
| Affect (e.g., pleasant, cooperative) | Withdrawn, intermittently tearful |
| Family group (e.g., who is family, who they live with) | Lives with spouse, no children, located far away from extended family |
| Education | All may be used |
| Level of health literacy | All may be used |
| Employment, if any - present and past, noting any current stresses | All may be used (including all enlisted and officer roles for military personnel) |
| Home/homeless - type of dwelling, number of stories, owned or rented | Lives in a home or apartment |
| Financial situation - any current stresses | All may be used |
| Insurance status (e.g., un/under/insured, public/private, HMO/PPO) | Insured |
| Habits (i.e., diet, exercise, caffeine, smoking, alcohol, drugs) | All may be used |
| Activities (i.e., hobbies, sports, clubs, friends) | All may be used |
| Typical day - what is the usual daily routine | All may be used |

| CASE INFORMATION | |
| --- | --- |
| Chief Concern: | When asked about what brings them to the ED, they will start the case by stating they are here with abdominal pain. |
| Additional Concerns: | Intimate partner violence: When asked about the onset of the pain or how it occurred, the patient should appear uncomfortable and provide minimal information unless they are directly asked if another person caused the abdominal pain or asked specifically about IPV. If the learner does not ask about IPV, the standard patient may note that the pain started during an argument with their spouse to prompt the learner. |
| THE PATIENT’S STORY: | “I started having abdominal pain a few days ago. It hurts pretty bad and I thought I could manage it at home, but I’ve been taking acetaminophen without any relief. It’s distracting me from work during the day and that started to make me worry more about the pain, so I came here.”  As the case continues, if asked directly about IPV:  “My spouse and I got into an argument. It happened really fast, but it got really heated and they pushed me. I fell onto a table onto my stomach, and that’s when all the pain started.”  If learner asks permission to further discuss IPV and asks about the events:  “I fell on the table, and I hit my stomach. I didn’t hit my head or lose consciousness, and they did not hit me anywhere else. They have only ever yelled at me before – they have never pushed me, this was the first time this has ever happened.”  If asked, the patient would like to discuss resources available to them and they are willing to meet with the Victim Advocate:  “Yeah, the past few days have been really overwhelming. I’m not sure where to go from here. I’d like to talk to the Victim Advocate about what my options are.” |
| HISTORY OF PRESENT ILLNESS: | |
| Onset (when; gradual or sudden) | Sudden (associated with trauma, if elicited by learner) |
| Setting (what was going on or where was patient when symptoms first noticed?) | At home, occurred after being pushed during argument with spouse |
| Duration (how long) | Several days |
| Time relationships (frequency, constant or intermittent) | Constant |
| Location | Epigastric |
| Radiation | Across top of abdomen to right and left upper quadrant |
| Quality | Aching |
| Amount | 5/10 rating of severity |
| Aggravated by what | Bending over |
| Relieved by what | Minimally relieved by acetaminophen |
| Associated with what | Mild nausea |
| Attitude (what does the patient think is the problem, and how do they feel about it) | Initially withdrawn to discuss onset as it is related to IPV, however if patient feels comfortable with learner and learner screens for IPV, they are willing to disclose IPV. |
| Overall course | Pain has not gone away, which is concerning the patient |
| REVIEW OF SYSTEMS: Significant positives and negatives | |
| Constitutional | No fevers, no chills, no weight loss |
| HEENT | No head strike or LoC, no sore throat, no rhinorrhea |
| Respiratory | No cough, no shortness of breath |
| Cardiovascular | No palpitations, no leg swelling |
| Gastrointestinal | No vomiting, no diarrhea; nausea is present |
| Genitourinary | No dysuria, no vaginal discharge or bleeding/penile discharge, no testicular pain if relevant |
| Skin | No rashes |
| Neuro | No numbness or tingling, no extremity weakness |
| Heme | No history of easy bruising; patient notes abdominal bruising present |
| Past medical history |  |
| Medication allergies (name and reaction) | None |
| Environmental allergies (name and reaction) | None |
| Illnesses | None |
| Vaccinations | Up to date |
| Surgeries | None |
| Accidents/injuries/trauma | Pushed into table several days ago, as described above |
| Hospitalization | No recent hospitalizations |
|  | |
| Inclusive sexual and reproductive history | |
| Sexual practices  Sexual partners  Protection: Use of safer sex practices  Use of birth control if appropriate  Risk of intimate partner violence | Sexually active with one partner, spouse.  All may be use for sexual partners, protection, birth control use.  Screens positive for intimate partner violence:   - Patient reports a history of verbal abuse by the spouse, with no history of physical abuse prior to a few days ago. They have access to the family car and finances without restriction. There are no children present in the home. They are located far away from their family since having to move for the military and have limited social support in the area.   The above script may be adjusted by SP and nurse educator during rehearsals. |
| OB/GYN history | Age of onset of menses – all may be used.  Age of menopause – all may be used.  Number of pregnancies – all may be used.  Number of live births – all may be used.  Number of miscarriages – all may be used.  Number of abortions – all may be used. |
| Medications | Prescription/dose/reason - None  Over the counter/dose/reason – Acetaminophen 650 mg every 6 hours as needed for pain at home since the abdominal pain started.  Herbs/supplements/dose/reason - None  Other: |
| Immunizations | Up to date on all immunizations |
| Tobacco products | All may be used |
| Alcohol | All may be used |
| Drugs | All may be used |
| Diet (describe) | 3 regular meals per day, no dietary restrictions |
| Exercise (describe) | Exercises 5 days per week |
| List any other important social history or information important to this case | See section on IPV above |
| Family history |  |
| Mother, father, siblings, grandparents, and other significant findings | No family history of bleeding disorders, otherwise all may be used |
|  |  |
| Physical Exam -  General: Seated on stretcher/chair, nontoxic but uncomfortable  HEENT: Normal  Neck: Normal  Cardiac: Normal  Pulmonary: Normal  Abdominal: Inspection reveals faint abdominal bruising. Abdomen is soft. Tenderness to palpation across epigastrium, no other areas of tenderness. Negative Murphy’s sign. No rebound tenderness.  Extremities: Normal  Neuro: Normal  Skin: Ecchymosis over epigastrium; no other rashes  Psych: Withdrawn, intermittently tearful | |
| PHYSICAL EXAM FINDINGS |  |
| 1. Written in layperson’s terms | Bruising over top of abdomen with tenderness over the area of bruising. |
| 1. General appearance - affect, appearance, position of patient at opening (i.e., sitting, lying down, holding abdomen, etc.) | Withdrawn affect, appears uncomfortable but not acutely ill, sitting on stretcher, holding arms over abdomen. |
| 1. Vital signs | Temp: 98.6 F, HR: 72 bpm, BP: 120/70 mm Hg, RR: 12 breaths/min, SpO2 99% on room air |
| 1. Specific findings and affect | Bruising over abdomen with tenderness, withdrawn and occasionally tearful affect |
| 1. Response to certain physical movements | Wince with palpation over epigastrium (top of stomach) |
|  |  |
| DIAGNOSIS AND DIFFERENTIAL |  |
| Diagnosis with support from positive and negative history and PE findings | The learner is not expected to report a primary diagnosis and differential with this learning exercise. |
| Differential with support from positive and negative history and PE findings | The learner is not expected to report a primary diagnosis and differential with this learning exercise. |
|  |  |
| MANAGEMENT OR DIAGNOSTIC PLAN | The learner is not expected to report a management or diagnostic plan with this learning exercise. |
|  |  |
| PROFESSIONALISM ISSUES OR CHALLENGES | 1. Failure to screen for intimate partner violence: It can be challenging for learners to breach the topic of intimate partner violence, often out of discomfort or fear of causing distress to the patient. We found that normalizing screening for intimate partner violence and creating a dedicated simulation exercise to this skill allowed learners to screen for IPV.  2. Failure to respond appropriately to patient’s emotional distress: Some learners may find it uncomfortable to respond to the patient’s distress or may focus entirely on the medical screening or attempt to physically comfort the patient without a trauma-informed approach. Reviewing common concerns that patients express when presenting after an assault and appropriate responses, as well as trauma-informed principles for history taking and patient interaction, was helpful in addressing this potential pitfall.  3. Using language that is not trauma-informed: Learners may try to ask questions using language that is not trauma-informed. For example, they may ask, “were you only pushed?” while attempting to elucidate if there are other injuries, thereby unintentionally minimizing the assault. We found it helpful to review approaches to trauma-informed language during the didactic session.  4. Not involving the victim advocate: We anticipate that some learners may forget to offer that the patient meet with the Victim Advocate. We found this a good learning opportunity to reinforce the importance of the Victim Advocate and the need to offer this resource to patients in the emergency department with concerns related to IPV. |

Appendix B: *MedEdPORTAL* Standardized Patient Case Development Tool – Case 2

Date: October 5^th^, 2022

Primary Case Author: Stefani Ramsey

Standardized Patient Educator: Stefani Ramsey

Name of Case: A Patient with Wrist Pain

Name of Educational Activity: Taking a Trauma-Informed History in Cases Related to Intimate Partner Violence

Patient Name: Sam Jones

Chief Complaint: Right Wrist Pain

Most Likely Diagnosis and Differential with Rationale from History and/or Physical Exam:

1. Distal wrist ligamentous strain - Suggested by mechanism of injury, location of pain, exam with ecchymosis and reproducible distal wrist tenderness without bony deformity.
2. Distal radius fracture – Suggested by location of pain, exam with ecchymosis and reproducible distal wrist tenderness.
3. Ulnar Styloid Fracture – Suggested by location of pain, exam with ecchymosis and reproducible distal wrist tenderness.

Domains: Check all that apply

✓ Professionalism

✓ Communication and Interpersonal Skills

✓ Medical History

- Physical Exam

✓ Shared Decision-Making

✓ Patient Education

- Clinical Reasoning
- Documentation
- Handoff
- Presentation

✓ Other: Trauma-Informed Care

Type and Level of Learner: Emergency medicine (EM) learners (medical students, residents, EM physician assistant fellows)

Case Objectives: Please list specific objectives for each of the domains you have checked above:

1. Identify concern for IPV.

2. Respond appropriately to the disclosure of IPV.

3. Establish a psychologically and physically safe environment for the patient using TIC principles.

4. Elucidate if the patient is willing to discuss next steps in managing IPV from the Emergency Department.

5. Offer that the patient may meet with the Victim Advocate.

6. Counsel the patient on resources available to them (if standardized patient is agreeable to further counseling).

| SETTING: | Emergency department examination room |
| --- | --- |
| PATIENT PROFILE: | |
| Age range | 18 - 50 |
| Religious/spiritual background | All may be used |
| Sex (e.g., male, female, intersex, transwoman, transman) | All may be used |
| Sexual orientation (e.g., heterosexual, lesbian, gay, bisexual, pansexual, queer, asexual) | All may be used |
| Gender expression (e.g., man, woman, genderqueer) | All may be used |
| Race and ethnicity | All may be used |
| Physical description (e.g., BMI, height range) | All may be used |
| Physical limitations | None |
| Patient appearance (e.g., disheveled, hospital gown, business casual, casual) | Casual civilian clothes |
| Moulage + location (e.g., none, bruises, scars, body piercing, tattoos) | Faint, circumferential ecchymosis over distal right wrist |
| Affect (e.g., pleasant, cooperative) | Withdrawn, intermittently tearful |
| Family group (e.g., who is family, who they live with) | Lives with spouse, no children, located far away from extended family |
| Education | All may be used |
| Level of health literacy | All may be used |
| Employment, if any - present and past, noting any current stresses | All may be used (this case was performed with the patient presenting as a civilian spouse, classified as a “dependent,” with civilian employment left up to the SP, but the patient may also be active duty). |
| Home/homeless - type of dwelling, number of stories, owned or rented | Lives in a home or apartment |
| Financial situation - any current stresses | All may be used |
| Insurance status (e.g., un/under/insured, public/private, HMO/PPO) | Insured |
| Habits (i.e., diet, exercise, caffeine, smoking, alcohol, drugs) | All may be used |
| Activities (i.e., hobbies, sports, clubs, friends) | All may be used |
| Typical day - what is the usual daily routine | All may be used |

| CASE INFORMATION | |
| --- | --- |
| Chief Concern: | When asked about what brings them to the ED, they will start the case by stating they are here with right wrist pain. |
| Additional Concerns: | Intimate partner violence: When asked about the onset of the pain or how it occurred, the patient should appear uncomfortable and provide minimal information unless they are directly asked if another person caused the wrist pain or asked specifically about IPV. If the learner does not ask about IPV, the standard patient may note that the pain started during an argument with their spouse to prompt the learner. |
| THE PATIENT’S STORY: | “I started having wrist pain a few days ago. It really hurts – I thought I could manage it at home with some pain medications, but it hasn’t gone away yet and that made me feel worried. I don’t really remember how it happened – I’m pretty accident prone.”  As the case continues, if asked directly about IPV:  “My spouse and I got into an argument. It happened so suddenly – they grabbed my wrist really hard and twisted it. It’s been hurting ever since then.”  If learner asks permission to further discuss IPV and asks about the events:  “They grabbed my wrist and twisted – it hurt when that happened. They did not hit me anywhere else. They have yelled at me before, but this is the first time it has ever gotten physical.”  If asked, the patient would like to discuss resources available to them and they are willing to meet with the Victim Advocate:  “Yes - I’m not sure where to go from here, and I need some help. I’d like to talk to the Victim Advocate about what my options are.” |
| HISTORY OF PRESENT ILLNESS: | |
| Onset (when; gradual or sudden) | Sudden (associated with trauma, if elicited by learner) |
| Setting (what was going on or where was patient when symptoms first noticed?) | At home, occurred after being grabbed during argument with spouse |
| Duration (how long) | Several days |
| Time relationships (frequency, constant or intermittent) | Constant |
| Location | Right wrist, distal wrist pain, circumferential |
| Radiation | Does not radiate |
| Quality | Aching, intermittently sharp when aggravated |
| Amount | 5/10 rating of severity |
| Aggravated by what | Moving the wrist |
| Relieved by what | Minimally relieved by acetaminophen |
| Associated with what | Mild swelling |
| Attitude (what does the patient think is the problem, and how do they feel about it) | Initially withdrawn to discuss onset as it is related to IPV, however if patient feels comfortable with learner and learner screens for IPV, they are willing to disclose IPV. |
| Overall course | Pain has not gone away, which is concerning the patient |
| REVIEW OF SYSTEMS: Significant positives and negatives | |
| Constitutional | No fevers, no chills, no weight loss |
| HEENT | No head strike or LoC, no sore throat, no rhinorrhea |
| Respiratory | No cough, no shortness of breath |
| Cardiovascular | No palpitations, no leg swelling |
| Gastrointestinal | No vomiting, no diarrhea |
| Genitourinary | No dysuria, no vaginal discharge or bleeding/penile discharge |
| Skin | No rashes |
| Neuro | No numbness or tingling of the affected extremity or elsewhere, no extremity weakness |
| Heme | No history of easy bruising, patient notes distal right wrist ecchymosis is present |
| Past medical history |  |
| Medication allergies (name and reaction) | None |
| Environmental allergies (name and reaction) | None |
| Illnesses | None |
| Vaccinations | Up to date |
| Surgeries | None |
| Accidents/injuries/trauma | Spouse grabbed wrist several days ago, as described above |
| Hospitalization | No recent hospitalizations |
|  | |
| Inclusive sexual and reproductive history | |
| Sexual practices  Sexual partners  Protection: Use of safer sex practices  Use of birth control if appropriate  Risk of intimate partner violence | Sexually active with one partner, spouse.  All may be use for sexual partners, protection, birth control use.  Screens positive for intimate partner violence:   - Patient reports a history of verbal abuse by the spouse, with no history of physical abuse prior to a few days ago. They have access to the family car and finances without restriction. There are no children present in the home. They are located far away from their family since having to move for the military and have limited social support in the area.   The above script may be adjusted by SP and nurse educator during rehearsals. |
| OB/GYN history | Age of onset of menses – all may be used.  Age of menopause – all may be used.  Number of pregnancies – all may be used.  Number of live births – all may be used.  Number of miscarriages – all may be used.  Number of abortions – all may be used. |
| Medications | Prescription/dose/reason - None  Over the counter/dose/reason – Acetaminophen 650 mg every 6 hours as needed for pain at home since the wrist pain started.  Herbs/supplements/dose/reason - None  Other: |
| Immunizations | Up to date on all immunizations |
| Tobacco products | All may be used |
| Alcohol | All may be used |
| Drugs | All may be used |
| Diet (describe) | 3 regular meals per day, no dietary restrictions |
| Exercise (describe) | Exercises 5 days per week |
| List any other important social history or information important to this case | N/A |
| Family history |  |
| Mother, father, siblings, grandparents, and other significant findings | No family history of bleeding disorders, otherwise all may be used |
|  |  |
| Physical Exam -  General: Seated on stretcher/chair, nontoxic but uncomfortable  HEENT: Normal  Neck: Normal  Cardiac: Normal  Pulmonary: Normal  Abdominal: Normal  Extremities: Inspection reveals faint, circumferential ecchymosis around the right distal wrist. The right distal wrist is tender to palpation over both the distal radius and ulna. There is no scaphoid tenderness. There is 5 out of 5 grip strength and wrist flexion and extension when compared to the contralateral side. Sensation is intact to light touch and 5 mm two-point discrimination in a radial, median, and ulnar distribution distal to the injury. There is a 2+ strong radial pulse present and brisk cap refill in all digits.  Neuro: Normal  Skin: Circumferential ecchymosis around right wrist, remainder of exam normal.  Psych: Withdrawn, intermittently tearful | |
| PHYSICAL EXAM FINDINGS |  |
| 1. Written in layperson’s terms | Bruising around wrist with tenderness over the area of bruising. |
| 1. General appearance - affect, appearance, position of patient at opening (i.e., sitting, lying down, holding abdomen, etc.) | Withdrawn affect, appears uncomfortable but not acutely ill, sitting on stretcher, cradling right wrist in left arm. |
| 1. Vital signs | Temp: 98.6 F, HR: 72 bpm, BP: 120/70 mm Hg, RR: 12 breaths/min, SpO2 99% on room air |
| 1. Specific findings and affect | Bruising over distal right wrist with tenderness, withdrawn and occasionally tearful affect |
| 1. Response to certain physical movements | Wince with palpation over the right wrist in all areas with bruising |
|  |  |
| DIAGNOSIS AND DIFFERENTIAL |  |
| Diagnosis with support from positive and negative history and PE findings | The learner is not expected to report a primary diagnosis and differential with this learning exercise. |
| Differential with support from positive and negative history and PE findings | The learner is not expected to report a primary diagnosis and differential with this learning exercise. |
|  |  |
| MANAGEMENT OR DIAGNOSTIC PLAN | The learner is not expected to report a management or diagnostic plan with this learning exercise. |
|  |  |
| PROFESSIONALISM ISSUES OR CHALLENGES | 1. Failure to screen for intimate partner violence: It can be challenging for learners to breach the topic of intimate partner violence, often out of discomfort or fear of causing distress to the patient. We found that normalizing screening for intimate partner violence and creating a dedicated simulation exercise to this skill allowed learners to screen for IPV.  2. Failure to respond appropriately to patient’s emotional distress: Some learners may find it uncomfortable to respond to the patient’s distress or may focus entirely on the medical screening or attempt to physically comfort the patient without a trauma-informed approach. Reviewing common concerns that patients express when presenting after an assault and appropriate responses, as well as trauma-informed principles for history taking and patient interaction, was helpful in addressing this potential mistake.  3. Using language that is not trauma-informed: Learners may try to ask questions using language that is not trauma-informed. For example, they may ask, “were you only grabbed?” while attempting to elucidate if there are other injuries, thereby unintentionally minimizing the assault. We found it helpful to review approaches to trauma-informed language during the didactic session.  4. Not involving the victim advocate: We anticipate that some learners may forget to offer that the patient meet with the Victim Advocate. We found this a good learning opportunity to reinforce the importance of the Victim Advocate and the need to offer this resource to patients in the emergency department with concerns related to IPV. |

Appendix B: *MedEdPORTAL* Standardized Patient Case Development Tool – Case 3

Date: October 5^th^, 2022

Primary Case Author: Stefani Ramsey

Standardized Patient Educator: Stefani Ramsey

Name of Case: A Soldier Presents with Rectal Pain

Name of Educational Activity: Taking a Trauma-Informed History in Cases Related to Sexual Assault

Patient Name: Sam Jones

Chief Complaint: Rectal Pain

Most Likely Diagnosis and Differential with Rationale from History and/or Physical Exam:

1. Anal laceration
2. Peri-anal hematoma
3. Rectal perforation

This differential diagnosis is all based on the reported history of potential trauma, suspected mechanism of injury, and the patient’s description of pain.

Domains: Check all that apply

✓ Professionalism

✓ Communication and Interpersonal Skills

✓ Medical History

- Physical Exam

✓ Shared Decision-Making

✓ Patient Education

- Clinical Reasoning
- Documentation
- Handoff
- Presentation

✓ Other: Trauma-Informed Care

Type and Level of Learner: Emergency medicine (EM) learners (medical students, residents, EM physician assistant fellows)

Case Objectives: Please list specific objectives for each of the domains you have checked above:

1. Identify that the patient may have experienced SA.

2. Respond appropriately to the disclosure of SA.

3. Establish a psychologically and physically safe environment for the patient.

4. Elucidate if the patient is willing to discuss next steps in managing SA from the Emergency Department.

5. Offer that the patient may meet with the Victim Advocate/Sexual Assault Medical Forensic Examiner.

6. Counsel the patient on resources available to them (if standardized patient is agreeable to further counseling).

| SETTING: | Emergency department examination room |
| --- | --- |
| PATIENT PROFILE: | |
| Age range | 18 - 45 |
| Religious/spiritual background | All may be used |
| Sex (e.g., male, female, intersex, transwoman, transman) | All may be used |
| Sexual orientation (e.g., heterosexual, lesbian, gay, bisexual, pansexual, queer, asexual) | All may be used |
| Gender expression (e.g., man, woman, genderqueer) | All may be used |
| Race and ethnicity | All may be used |
| Physical description (e.g., BMI, height range) | All may be used |
| Physical limitations | None |
| Patient appearance (e.g., disheveled, hospital gown, business casual, casual) | Sweatpants, sweatshirt with hood pulled over head |
| Moulage + location (e.g., none, bruises, scars, body piercing, tattoos) | None |
| Affect (e.g., pleasant, cooperative) | Withdrawn, intermittently appears agitated but re-directable |
| Family group (e.g., who is family, who they live with) | Lives in military barracks, not married, no children |
| Education | All may be used |
| Level of health literacy | All may be used |
| Employment, if any - present and past, noting any current stresses | In this case, the service member was enlisted and lived in the barracks. The patient may be played by any job or rank in the military.  If educators choose to utilize this case with the SP playing a civilian, any employment may be used. |
| Home/homeless - type of dwelling, number of stories, owned or rented | Lives in military barracks |
| Financial situation - any current stresses | All may be used |
| Insurance status (e.g., un/under/insured, public/private, HMO/PPO) | Insured |
| Habits (i.e., diet, exercise, caffeine, smoking, alcohol, drugs) | Patient occasionally drinks with friends on the weekend. Otherwise, all may be used. |
| Activities (i.e., hobbies, sports, clubs, friends) | All may be used |
| Typical day - what is the usual daily routine | All may be used |

| CASE INFORMATION | |
| --- | --- |
| Chief Concern: | When asked about what brings them to the ED, they will start the case by stating they attended a party the night before and woke up the next morning with rectal pain, having no recollection of the prior night. |
| Additional Concerns: | Sexual assault: When the learner screens for sexual assault, the SP will disclose they are concerned that they may have been assaulted last night but cannot remember the events. |
| THE PATIENT’S STORY: | “I went to a party off post last night. I had a few drinks, but I don’t remember much. I woke up this morning and have no idea what happened – my rectal area really hurts, and I’m really freaked out.”  As the case continues, the learner screens for SA:  “I’m scared that I was sexually assaulted.”  If learner asks permission to further discuss SA and asks about the events:  “I really can’t remember anything. I wish I could tell you more.”  If asked, the patient would like to discuss resources available to them and the different reporting options, but expresses apprehension:  “I don’t know if I want to report this – what are the reporting options? Will my unit find out?”  After reviewing reporting options, the patient states:  “I think I want to talk a forensic nurse and Victim Advocate.” |
| HISTORY OF PRESENT ILLNESS: | |
| Onset (when; gradual or sudden) | Woke up with pain |
| Setting (what was going on or where was patient when symptoms first noticed?) | Patient woke up in the barracks |
| Duration (how long) | Since waking up this morning |
| Time relationships (frequency, constant or intermittent) | Constant |
| Location | Rectal pain |
| Radiation | Does not radiate |
| Quality | Aching |
| Amount | 5/10 rating of severity |
| Aggravated by what | Sitting |
| Relieved by what | Nothing |
| Associated with what | Nothing |
| Attitude (what does the patient think is the problem, and how do they feel about it) | The patient is feeling very anxious and distressed by the symptoms they are having. |
| Overall course | The pain and circumstances concerned the patient this morning, prompting them to seek care in the ED. |
| REVIEW OF SYSTEMS: Significant positives and negatives | |
| Constitutional | No fevers, no chills, no weight loss |
| HEENT | No known head strike or LoC, no sore throat, no rhinorrhea |
| Respiratory | No cough, no shortness of breath |
| Cardiovascular | No palpitations, no leg swelling |
| Gastrointestinal | No vomiting, no diarrhea, no rectal bleeding |
| Genitourinary | No dysuria, no vaginal discharge or bleeding/penile discharge, no testicular pain if applicable |
| Skin | No rashes |
| Neuro | No numbness or tingling of the extremities, no extremity weakness |
| Heme | No history of easy bruising |
| Past medical history |  |
| Medication allergies (name and reaction) | None |
| Environmental allergies (name and reaction) | None |
| Illnesses | None |
| Vaccinations | Up to date on all childhood and adult vaccinations |
| Surgeries | None |
| Accidents/injuries/trauma | Unclear circumstances at party, as explained above |
| Hospitalization | No recent hospitalizations |
|  | |
| Inclusive sexual and reproductive history | |
| Sexual practices  Sexual partners  Protection: Use of safer sex practices  Use of birth control if appropriate  Risk of intimate partner violence | Not currently sexually active  The above script may be adjusted by SP and nurse educator during rehearsals. |
| OB/GYN history | Age of onset of menses – all may be used.  Age of menopause – all may be used.  Number of pregnancies – all may be used.  Number of live births – all may be used.  Number of miscarriages – all may be used.  Number of abortions – all may be used. |
| Medications | Prescription/dose/reason - None  Over the counter/dose/reason - None  Herbs/supplements/dose/reason - None  Other: |
| Immunizations | Up to date on all immunizations |
| Tobacco products | All may be used |
| Alcohol | All may be used |
| Drugs | All may be used |
| Diet (describe) | 3 regular meals per day, no dietary restrictions |
| Exercise (describe) | Exercises 5 days per week |
| List any other important social history or information important to this case | N/A |
| Family history |  |
| Mother, father, siblings, grandparents, and other significant findings | No family history of bleeding disorders, otherwise all may be used |
|  |  |
| Physical Exam -  General: Standing in corner of room, appears anxious  HEENT: Normal  Neck: Normal  Cardiac: Normal  Pulmonary: Normal  Abdominal: Normal  Genitourinary Exam/Rectal Exam: Deferred  Neuro: Normal  Skin: Normal  Psych: Withdrawn, intermittently tearful and distraught | |
| PHYSICAL EXAM FINDINGS |  |
| 1. Written in layperson’s terms | Sensitive exams deferred (purpose of exercise is to focus on a trauma informed history) |
| 1. General appearance - affect, appearance, position of patient at opening (i.e., sitting, lying down, holding abdomen, etc.) | Withdrawn affect, appears uncomfortable but not acutely ill, standing in corner, intermittently distraught |
| 1. Vital signs | Temp: 98.6 F, HR: 72 bpm, BP: 120/70 mm Hg, RR: 12 breaths/min, SpO2 99% on room air |
| 1. Specific findings and affect | No specific exam findings |
| 1. Response to certain physical movements | Discomfort with sitting |
|  |  |
| DIAGNOSIS AND DIFFERENTIAL |  |
| Diagnosis with support from positive and negative history and PE findings | The learner is not expected to report a primary diagnosis and differential with this learning exercise. |
| Differential with support from positive and negative history and PE findings | The learner is not expected to report a primary diagnosis and differential with this learning exercise. |
|  |  |
| MANAGEMENT OR DIAGNOSTIC PLAN | The learner is not expected to report a management or diagnostic plan with this learning exercise. |
|  |  |
| PROFESSIONALISM ISSUES OR CHALLENGES | 1. Inadequate history: Many learners may be uncomfortable asking for pertinent details about the assault for this patient because they appear anxious, tearful, and just experienced a traumatic event. We found it helpful to review the information that we should obtain for a comprehensive medical evaluation prior to the simulation during a didactic session so that learners know how to balance asking the right amount of information from patients who are experiencing distress.  2. Failure to respond appropriately to patient’s emotional distress: Some learners may find it uncomfortable to respond to the patient’s distress or may focus entirely on the medical screening or attempt to physically comfort the patient without a trauma-informed approach. Reviewing common concerns that patients express when presenting after an assault and appropriate responses, as well as trauma-informed principles for history taking and patient interaction, may be helpful to avoid this potential mistake.  3. Using language that is not trauma-informed: Learners may try to ask questions using language that is not trauma-informed. For example, they may ask, “so they only punched you?” while attempting to elucidate details about the event, thereby unintentionally minimizing the patient’s experience. We found it helpful to review approaches to trauma-informed language during the didactic session.  4. Not offering the Victim Advocate/SAMFE: We anticipate that some learners may forget to offer that the patient meet with the Victim Advocate/SAMFE. We found this a good learning opportunity to reinforce the importance of knowing what resources are available to patients and providers to provide the patient with comprehensive evaluation and treatment options. |

Appendix B: *MedEdPORTAL* Standardized Patient Case Development Tool – Case 4

Date: October 5^th^, 2022

Primary Case Author: Stefani Ramsey

Standardized Patient Educator: Stefani Ramsey

Name of Case: A Soldier Presents after a Sexual Assault

Name of Educational Activity: Taking a Trauma-Informed History in Cases Related to Sexual Assault

Patient Name: Sam Jones

Chief Complaint: Sexual Assault

Most Likely Diagnosis and Differential with Rationale from History and/or Physical Exam:

1. Sexual Assault
2. Exposure to Sexually Transmitted Infection
3. Exposure to Unintended Pregnancy

This differential diagnosis is all based on the reported history of a sexual assault.

Domains: Check all that apply

✓ Professionalism

✓ Communication and Interpersonal Skills

✓ Medical History

- Physical Exam

✓ Shared Decision-Making

✓ Patient Education

- Clinical Reasoning
- Documentation
- Handoff
- Presentation

✓ Other: Trauma-Informed Care

Type and Level of Learner: Emergency medicine (EM) learners (medical students, residents, EM physician assistant fellows)

Case Objectives: Please list specific objectives for each of the domains you have checked above:

1. Identify that the patient may have experienced SA.

2. Respond appropriately to the disclosure of SA.

3. Establish a psychologically and physically safe environment for the patient.

4. Elucidate if the patient is willing to discuss next steps in managing SA from the Emergency Department.

5. Offer that the patient may meet with the Victim Advocate/Sexual Assault Medical Forensic Examiner.

6. Counsel the patient on resources available to them (if standardized patient is agreeable to further counseling).

| SETTING: | Emergency department examination room |
| --- | --- |
| PATIENT PROFILE: | |
| Age range | 18 - 45 |
| Religious/spiritual background | All may be used |
| Sex (e.g., male, female, intersex, transwoman, transman) | Female |
| Sexual orientation (e.g., heterosexual, lesbian, gay, bisexual, pansexual, queer, asexual) | All may be used |
| Gender expression (e.g., man, woman, genderqueer) | All may be used |
| Race and ethnicity | All may be used |
| Physical description (e.g., BMI, height range) | All may be used |
| Physical limitations | None |
| Patient appearance (e.g., disheveled, hospital gown, business casual, casual) | Casual clothes |
| Moulage + location (e.g., none, bruises, scars, body piercing, tattoos) | None |
| Affect (e.g., pleasant, cooperative) | Appears anxious, intermittently tearful |
| Family group (e.g., who is family, who they live with) | Lives in military barracks, lives adjacent to members in their unit, not married, no children |
| Education | All may be used |
| Level of health literacy | All may be used |
| Employment, if any - present and past, noting any current stresses | In this case, the service member was enlisted and lived in the barracks. |
| Home/homeless - type of dwelling, number of stories, owned or rented | Lives in military barracks |
| Financial situation - any current stresses | All may be used |
| Insurance status (e.g., un/under/insured, public/private, HMO/PPO) | Insured |
| Habits (i.e., diet, exercise, caffeine, smoking, alcohol, drugs) | Patient occasionally drinks with friends on the weekend. Otherwise, all may be used. |
| Activities (i.e., hobbies, sports, clubs, friends) | All may be used |
| Typical day - what is the usual daily routine | All may be used |

| CASE INFORMATION | |
| --- | --- |
| Chief Concern: | When asked about what brings them to the ED, they will report that they experienced a sexual assault. |
| Additional Concerns: | The patient also notes abdominal pain. |
| THE PATIENT’S STORY: | “A few hours ago, I was leaving my room when a guy was waiting when I opened the door and pushed me back in. I tried to scream but he punched me in the stomach. After that, he held me down and assaulted me.”  If learner asks permission to further discuss SA and asks about the events:  “It’s hard to remember. It all happened so fast. I think they used a condom but I don’t know. My stomach and genital area really hurt.”  If learner specifically asks about choking or strangling: “No, I was not choked or strangled.”  If asked, the patient would like to discuss resources available to them and the different reporting options, but expresses apprehension:  “I don’t know if I want to report this – what are the reporting options? Will my unit find out?”  After reviewing reporting options, the patient states:  “I think I want to talk a forensic nurse and Victim Advocate.” |
| HISTORY OF PRESENT ILLNESS: | |
| Onset (when; gradual or sudden) | Abdominal pain started immediately after the assault |
| Setting (what was going on or where was patient when symptoms first noticed?) | Barracks |
| Duration (how long) | 2 hours |
| Time relationships (frequency, constant or intermittent) | Constant |
| Location | Upper and lower abdominal pain |
| Radiation | Does not radiate |
| Quality | Aching |
| Amount | 5/10 rating of severity |
| Aggravated by what | Moving |
| Relieved by what | Nothing |
| Associated with what | Nausea |
| Attitude (what does the patient think is the problem, and how do they feel about it) | The patient expresses feeling very anxious and distressed by the assault. |
| Overall course | The patient came to the ED once a friend was able to pick them up from the barracks and drive them. |
| REVIEW OF SYSTEMS: Significant positives and negatives | |
| Constitutional | No fevers, no chills, no weight loss |
| HEENT | No known head strike or LoC, no sore throat, no rhinorrhea |
| Respiratory | No cough, no shortness of breath |
| Cardiovascular | No palpitations, no leg swelling |
| Gastrointestinal | No vomiting, no diarrhea, no rectal bleeding or pain; +nausea present |
| Genitourinary | Pain in the vaginal introitus. No dysuria, no vaginal discharge, no vaginal bleeding. |
| Skin | No rashes |
| Neuro | No numbness or tingling of the extremities, no extremity weakness |
| Heme | No history of easy bruising |
| Past medical history |  |
| Medication allergies (name and reaction) | None |
| Environmental allergies (name and reaction) | None |
| Illnesses | None |
| Vaccinations | Up to date on all childhood and adult vaccinations. |
| Surgeries | None |
| Accidents/injuries/trauma | Punched in the center of the abdomen once. Forced vaginal penetration during assault. |
| Hospitalization | No recent hospitalization |
|  | |
| Inclusive sexual and reproductive history | |
| Sexual practices  Sexual partners  Protection: Use of safer sex practices  Use of birth control if appropriate  Risk of intimate partner violence | Not currently sexually active  The above script may be adjusted by SP and nurse educator during rehearsals. |
| OB/GYN history | Age of onset of menses – all may be used.  Age of menopause – all may be used.  Number of pregnancies – all may be used.  Number of live births – all may be used.  Number of miscarriages – all may be used.  Number of abortions – all may be used. |
| Medications | Prescription/dose/reason - None  Over the counter/dose/reason - None  Herbs/supplements/dose/reason - None  Other: |
| Immunizations | Up to date on all immunizations |
| Tobacco products | All may be used |
| Alcohol | All may be used |
| Drugs | All may be used |
| Diet (describe) | 3 regular meals per day, no dietary restrictions |
| Exercise (describe) | Exercises 5 days per week |
| List any other important social history or information important to this case | N/A |
| Family history |  |
| Mother, father, siblings, grandparents, and other significant findings | No family history of bleeding disorders, otherwise all may be used |
|  |  |
| Physical Exam -  General: Sitting on ED stretcher, appears anxious  HEENT: Normal, no external signs of trauma  Neck: Normal, no petechiae, no ecchymosis, no bruit  Cardiac: Normal  Pulmonary: Normal  Abdominal: Mild tenderness diffusely without rebound, guarding, or ecchymosis.  Genitourinary Exam/Rectal Exam: Deferred  Neuro: Normal  Skin: Normal  Psych: Withdrawn, intermittently tearful and distraught | |
| PHYSICAL EXAM FINDINGS |  |
| 1. Written in layperson’s terms | Sensitive exams deferred (purpose of exercise is to focus on a trauma informed history) |
| 1. General appearance - affect, appearance, position of patient at opening (i.e., sitting, lying down, holding abdomen, etc.) | Withdrawn affect, appears uncomfortable but not acutely ill, standing in corner, intermittently distraught |
| 1. Vital signs | Temp: 98.6 F, HR: 72 bpm, BP: 120/70 mm Hg, RR: 12 breaths/min, SpO2 99% on room air |
| 1. Specific findings and affect | Diffuse mild abdominal tenderness |
| 1. Response to certain physical movements | Discomfort with sitting |
|  |  |
| DIAGNOSIS AND DIFFERENTIAL |  |
| Diagnosis with support from positive and negative history and PE findings | The learner is not expected to report a primary diagnosis and differential with this learning exercise. |
| Differential with support from positive and negative history and PE findings | The learner is not expected to report a primary diagnosis and differential with this learning exercise. |
|  |  |
| MANAGEMENT OR DIAGNOSTIC PLAN | The learner is not expected to report a management or diagnostic plan with this learning exercise. |
|  |  |
| PROFESSIONALISM ISSUES OR CHALLENGES | 1. Inadequate history: Many learners may be uncomfortable asking for pertinent details about the assault for this patient because they appear anxious, tearful, and just experienced a traumatic event. We found it helpful to review the information that we should obtain for a comprehensive medical evaluation prior to the simulation during a didactic session so that learners know how to balance asking the right amount of information from patients who are experiencing distress.  2. Failure to respond appropriately to patient’s emotional distress: Some learners may find it uncomfortable to respond to the patient’s distress or may focus entirely on the medical screening or attempt to physically comfort the patient without a trauma-informed approach. Reviewing common concerns that patients express when presenting after an assault and appropriate responses, as well as trauma-informed principles for history taking and patient interaction, may be helpful to avoid this potential mistake.  3. Using language that is not trauma-informed: Learners may try to ask questions using language that is not trauma-informed. For example, they may ask, “so they only punched you?” while attempting to elucidate details about the event, thereby unintentionally minimizing the patient’s experience. We found it helpful to review approaches to trauma-informed language during the didactic session.  4. Not offering the Victim Advocate/SAMFE: We anticipate that some learners may forget to offer that the patient meet with the Victim Advocate/SAMFE. We found this a good learning opportunity to reinforce the importance of knowing what resources are available to patients and providers to provide the patient with comprehensive evaluation and treatment options. |
