# Supplementary material for: Trauma-Informed Care for Intimate Partner Violence and Sexual Assault: Simulated Participant Cases for Emergency Medicine Learners
Source: MedEdPORTAL. 2025 Feb 25;21:11500. doi: 10.15766/mep_2374-8265.11500 (PMC11850505; doi:10.15766/mep_2374-8265.11500)
Supplement: Supplementary file 1 — Didactic Lecture.pptxSP Case Development Tool.docxCritical Actions Checklist.docxPre- and Postcurriculum Self-Assessments.docx [file mep_2374-8265.11500-s001.zip › C. Critical Actions Checklist.docx]

**Appendix C: Critical Actions Checklist**

Provide to SPs, observers, and faculty prior to starting simulation event to encourage structured feedback delivered to learner after simulation. May be adapted to assess and measure learner performance.

**Intimate Partner Violence (IPV) Cases Critical Actions Checklist:**

1. Identify concern for IPV.
2. Respond appropriately to the disclosure of IPV.
3. Establish a psychologically and physically safe environment for the patient using TIC principles.
4. Elucidate if the patient is willing to discuss next steps in managing IPV from the Emergency Department.
5. Offer that the patient may meet with the Victim Advocate.
6. Counsel the patient on resources available to them (if standardized patient is agreeable to further counseling).

**Sexual Assault (SA) Cases Critical Actions Checklist:**

1. Identify that the patient may have experienced SA.
2. Respond appropriately to the disclosure of SA.
3. Establish a psychologically and physically safe environment for the patient.
4. Elucidate if the patient is willing to discuss next steps in managing SA from the Emergency Department.
5. Offer that the patient may meet with the Victim Advocate/Sexual Assault Medical Forensic Examiner.
6. Counsel the patient on resources available to them (if standardized patient is agreeable to further counseling).
