# Supplementary material for: Trauma-Informed Care for Intimate Partner Violence and Sexual Assault: Simulated Participant Cases for Emergency Medicine Learners
Source: MedEdPORTAL. 2025 Feb 25;21:11500. doi: 10.15766/mep_2374-8265.11500 (PMC11850505; doi:10.15766/mep_2374-8265.11500)
Supplement: Supplementary file 1 — Didactic Lecture.pptxSP Case Development Tool.docxCritical Actions Checklist.docxPre- and Postcurriculum Self-Assessments.docx [file mep_2374-8265.11500-s001.zip › D. Pre- and Postcurriculum Self-Assessments.docx]

**Appendix D: Pre and Post Curriculum Self-Assessments**

**User Guide**

Timing: <5 minutes per assessment

Use: Measure change in learner self-assessed competency in caring for patients experiencing intimate partner violence (IPV) or sexual assault (SA) pre and post curriculum

Administration: Provide a printed pre-curriculum assessment to each learner prior to the didactic lecture. A printed post-curriculum assessment should be provided following completion of the simulation exercise.

Potential modifications:

- Recommend adding a question to the post-assessment to track which case each learner completed
- Consider adding a code or other de-identified marker to track individual learner pre and post self-assessments

Some questions in this assessment are adapted from the PREMIS Tool, which has been developed and utilized to measure physician readiness in managing IPV. The authors of the tool made it available for use by educators:

Short L, Alpert E, Harris J, Suprenant Z. PREMIS: A Comprehensive and Reliable Tool for Measuring Physician Readiness to Manage IPV. Am J Prev Med. 2006;30(2):173-180. doi:10.1016/j.amepre.2005.10.009

**Pre-Curriculum Self Assessment**

| 1. Please indicate your level of training: | Medical Student | PGY1 | PGY2 | PGY3 | EM-PA |
| --- | --- | --- | --- | --- | --- |

1. For each of the following statements related to Intimate Partner Violence (IPV), please indicate your response:

(1 = Strongly Disagree, 2 - Disagree, 3 - Neutral, 4 - Agree, 5 - Strongly Agree)

| 1. I feel comfortable discussing IPV with my patients | 1 | 2 | 3 | 4 | 5 |
| --- | --- | --- | --- | --- | --- |
| 1. I can appropriately respond to disclosures of IPV | 1 | 2 | 3 | 4 | 5 |
| 1. I am able to gather and record the necessary information to care for patients presenting with IPV | 1 | 2 | 3 | 4 | 5 |
| 1. I understand and utilize strategies to help patients presenting with IPV-related concerns feel physically and psychologically safe | 1 | 2 | 3 | 4 | 5 |
| 1. I recognize how biases and cultural norms may influence IPV-related patient encounters | 1 | 2 | 3 | 4 | 5 |
| 1. I feel comfortable providing counseling on therapeutic interventions for patients who have experienced IPV | 1 | 2 | 3 | 4 | 5 |

1. For each of the following statements related to Sexual Assault (SA), please indicate your response:

(1 = Strongly Disagree, 2 - Disagree, 3 - Neutral, 4 - Agree, 5 - Strongly Agree)

| 1. I feel comfortable discussing SA with my patients | 1 | 2 | 3 | 4 | 5 |
| --- | --- | --- | --- | --- | --- |
| 1. I can appropriately respond to disclosures of SA | 1 | 2 | 3 | 4 | 5 |
| 1. I am able to gather and record the necessary information to care for patients presenting with SA | 1 | 2 | 3 | 4 | 5 |
| 1. I understand and utilize strategies to help patients presenting with SA-related concerns feel physically and psychologically safe | 1 | 2 | 3 | 4 | 5 |
| 1. I recognize how biases and cultural norms may influence SA-related patient encounters | 1 | 2 | 3 | 4 | 5 |
| 1. I feel comfortable providing counseling on therapeutic interventions for patients who have experienced SA | 1 | 2 | 3 | 4 | 5 |

**Post-Curriculum Self Assessment**

| 1. Please indicate your level of training: | Medical Student | PGY1 | PGY2 | PGY3 | EM-PA |
| --- | --- | --- | --- | --- | --- |

1. For each of the following statements related to Intimate Partner Violence (IPV), please indicate your response:

(1 = Strongly Disagree, 2 - Disagree, 3 - Neutral, 4 - Agree, 5 - Strongly Agree)

| 1. I feel comfortable discussing IPV with my patients | 1 | 2 | 3 | 4 | 5 |
| --- | --- | --- | --- | --- | --- |
| 1. I can appropriately respond to disclosures of IPV | 1 | 2 | 3 | 4 | 5 |
| 1. I am able to gather and record the necessary information to care for patients presenting with IPV | 1 | 2 | 3 | 4 | 5 |
| 1. I understand and utilize strategies to help patients presenting with IPV-related concerns feel physically and psychologically safe | 1 | 2 | 3 | 4 | 5 |
| 1. I recognize how biases and cultural norms may influence IPV-related patient encounters | 1 | 2 | 3 | 4 | 5 |
| 1. I feel comfortable providing counseling on therapeutic interventions for patients who have experienced IPV | 1 | 2 | 3 | 4 | 5 |

1. For each of the following statements related to Sexual Assault (SA), please indicate your response:

(1 = Strongly Disagree, 2 - Disagree, 3 - Neutral, 4 - Agree, 5 - Strongly Agree)

| 1. I feel comfortable discussing SA with my patients | 1 | 2 | 3 | 4 | 5 |
| --- | --- | --- | --- | --- | --- |
| 1. I can appropriately respond to disclosures of SA | 1 | 2 | 3 | 4 | 5 |
| 1. I am able to gather and record the necessary information to care for patients presenting with SA | 1 | 2 | 3 | 4 | 5 |
| 1. I understand and utilize strategies to help patients presenting with SA-related concerns feel physically and psychologically safe | 1 | 2 | 3 | 4 | 5 |
| 1. I recognize how biases and cultural norms may influence SA-related patient encounters | 1 | 2 | 3 | 4 | 5 |
| 1. I feel comfortable providing counseling on therapeutic interventions for patients who have experienced SA | 1 | 2 | 3 | 4 | 5 |

1. Please indicate your response regarding this training:

(1 = Strongly Disagree, 2 - Disagree, 3 - Neutral, 4 - Agree, 5 - Strongly Agree)

| 1. Didactic-based learning is an effective way to improve clinical skills in IPV or SA-related encounters | 1 | 2 | 3 | 4 | 5 |
| --- | --- | --- | --- | --- | --- |
| 1. Simulation-based learning is an effective way to improve clinical skills in IPV or SA -related encounters | 1 | 2 | 3 | 4 | 5 |
| 1. IPV or SA-related simulation cases are realistic to what ED providers may encounter in the department | 1 | 2 | 3 | 4 | 5 |

1. Please provide any recommendations or comments for how training on this topic can be improved:

|  |
| --- |
