# Supplementary material for: Trauma-Informed Care for Intimate Partner Violence and Sexual Assault: Simulated Participant Cases for Emergency Medicine Learners
Source: MedEdPORTAL. 2025 Feb 25;21:11500. doi: 10.15766/mep_2374-8265.11500 (PMC11850505; doi:10.15766/mep_2374-8265.11500)
Supplement: Supplementary file 1 — Didactic Lecture.pptxSP Case Development Tool.docxCritical Actions Checklist.docxPre- and Postcurriculum Self-Assessments.docx [file mep_2374-8265.11500-s001.zip › A. Didactic Lecture.pptx]

## Slide 1
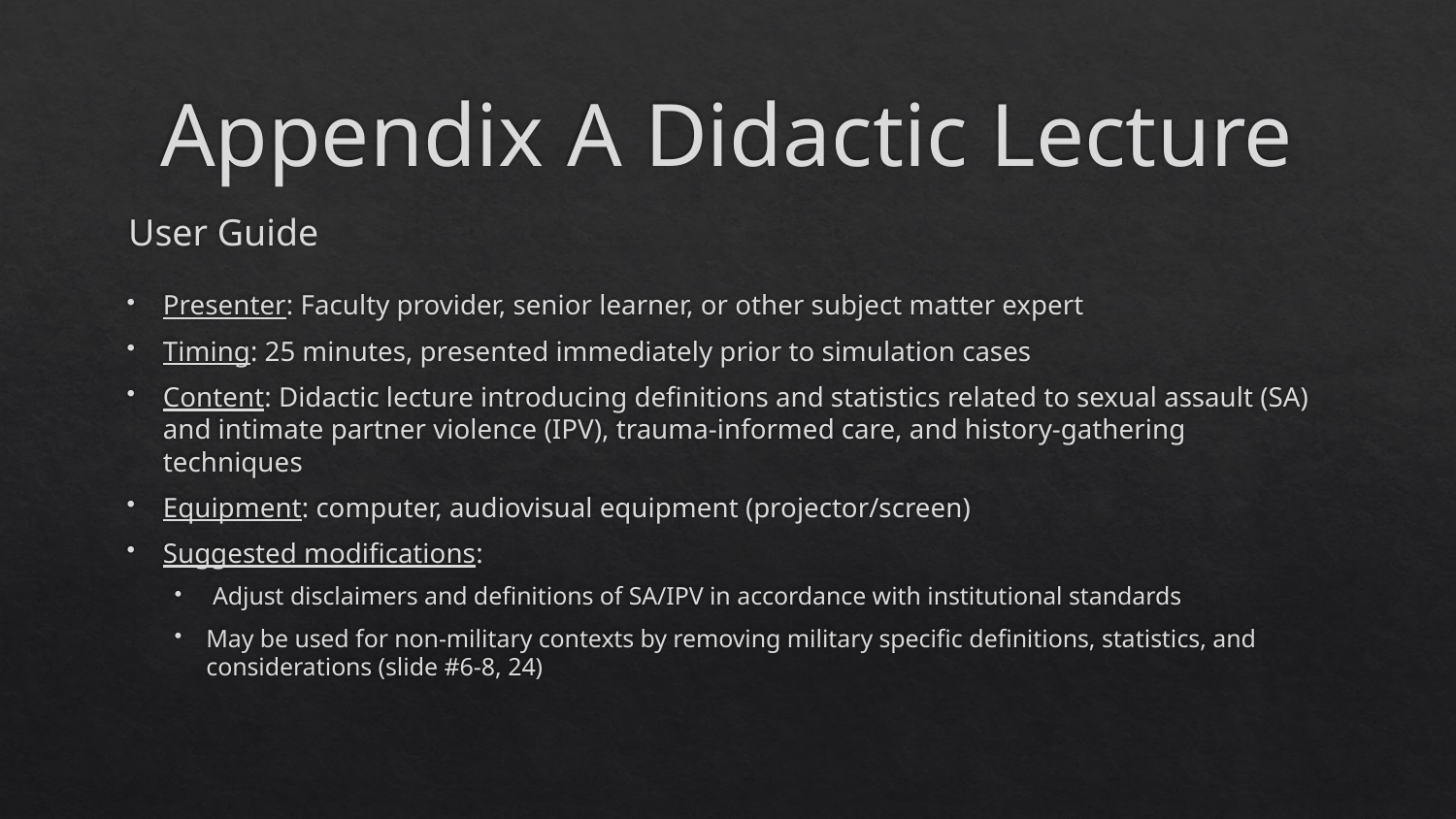

# Appendix A Didactic Lecture
User Guide
Presenter: Faculty provider, senior learner, or other subject matter expert
Timing: 25 minutes, presented immediately prior to simulation cases
Content: Didactic lecture introducing definitions and statistics related to sexual assault (SA) and intimate partner violence (IPV), trauma-informed care, and history-gathering techniques
Equipment: computer, audiovisual equipment (projector/screen)
Suggested modifications:
 Adjust disclaimers and definitions of SA/IPV in accordance with institutional standards
May be used for non-military contexts by removing military specific definitions, statistics, and considerations (slide #6-8, 24)

## Slide 2
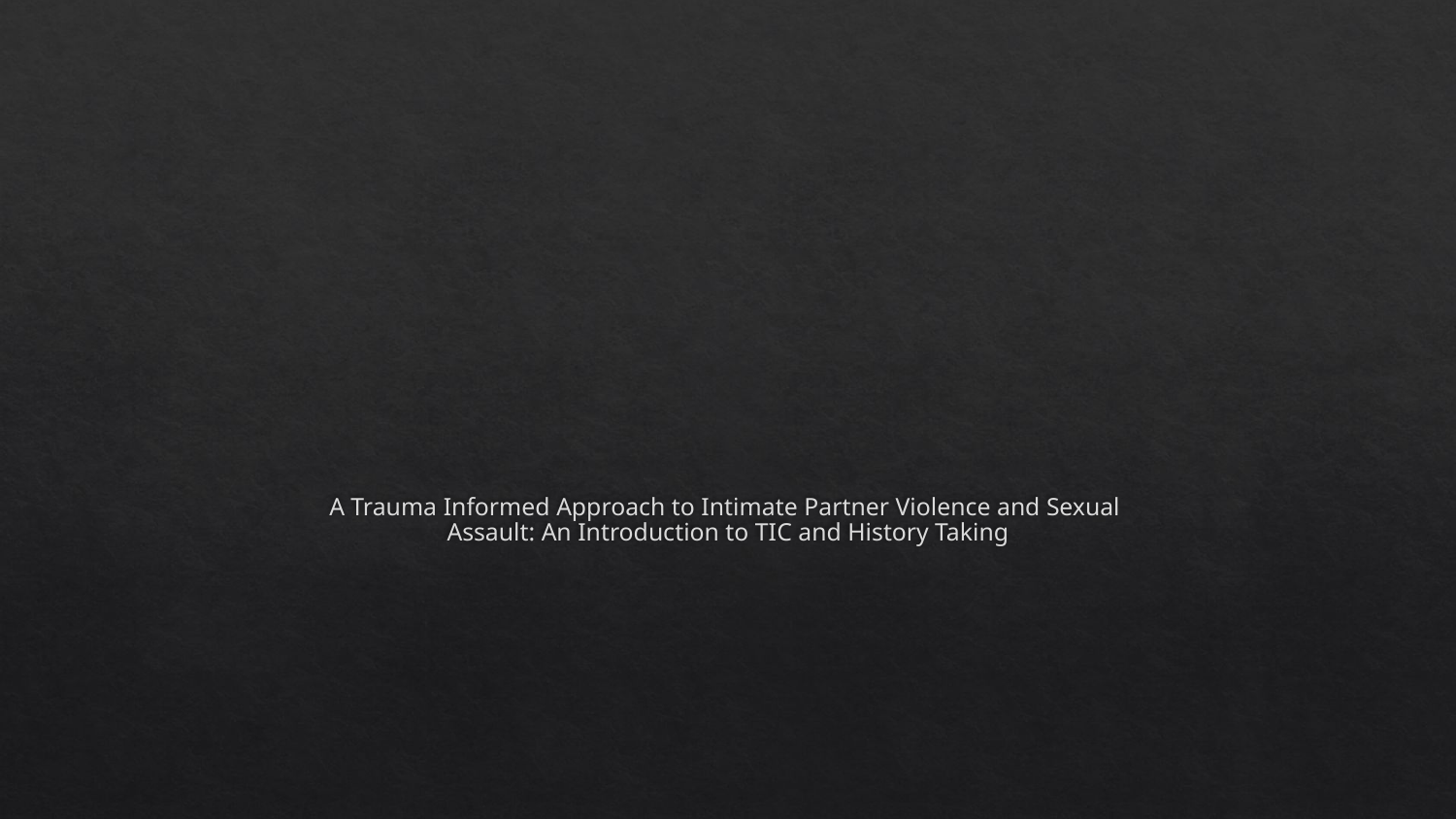

# A Trauma Informed Approach to Intimate Partner Violence and Sexual Assault: An Introduction to TIC and History Taking

## Slide 3
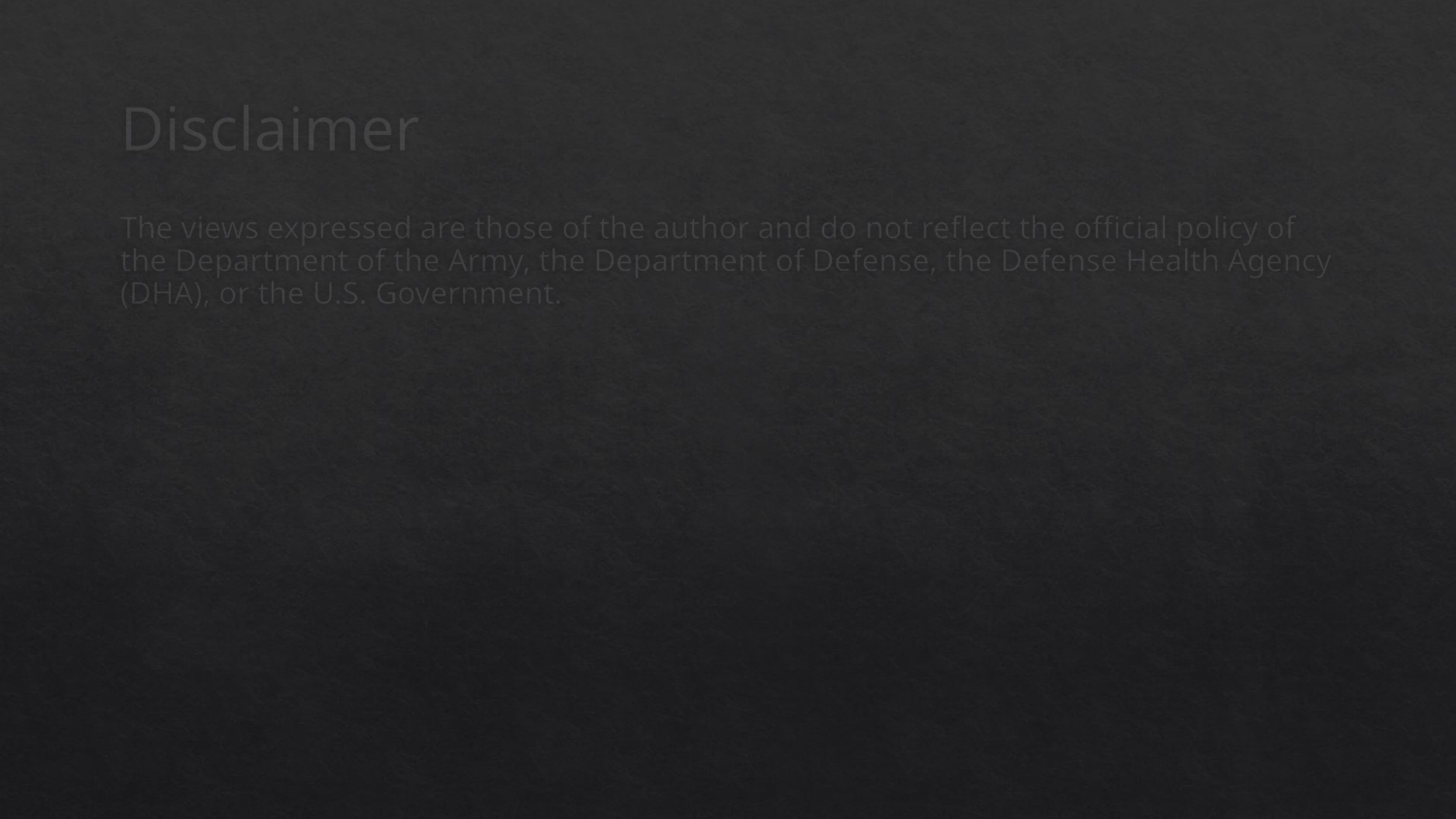

# Disclaimer
The views expressed are those of the author and do not reflect the official policy of the Department of the Army, the Department of Defense, the Defense Health Agency (DHA), or the U.S. Government.

## Slide 4
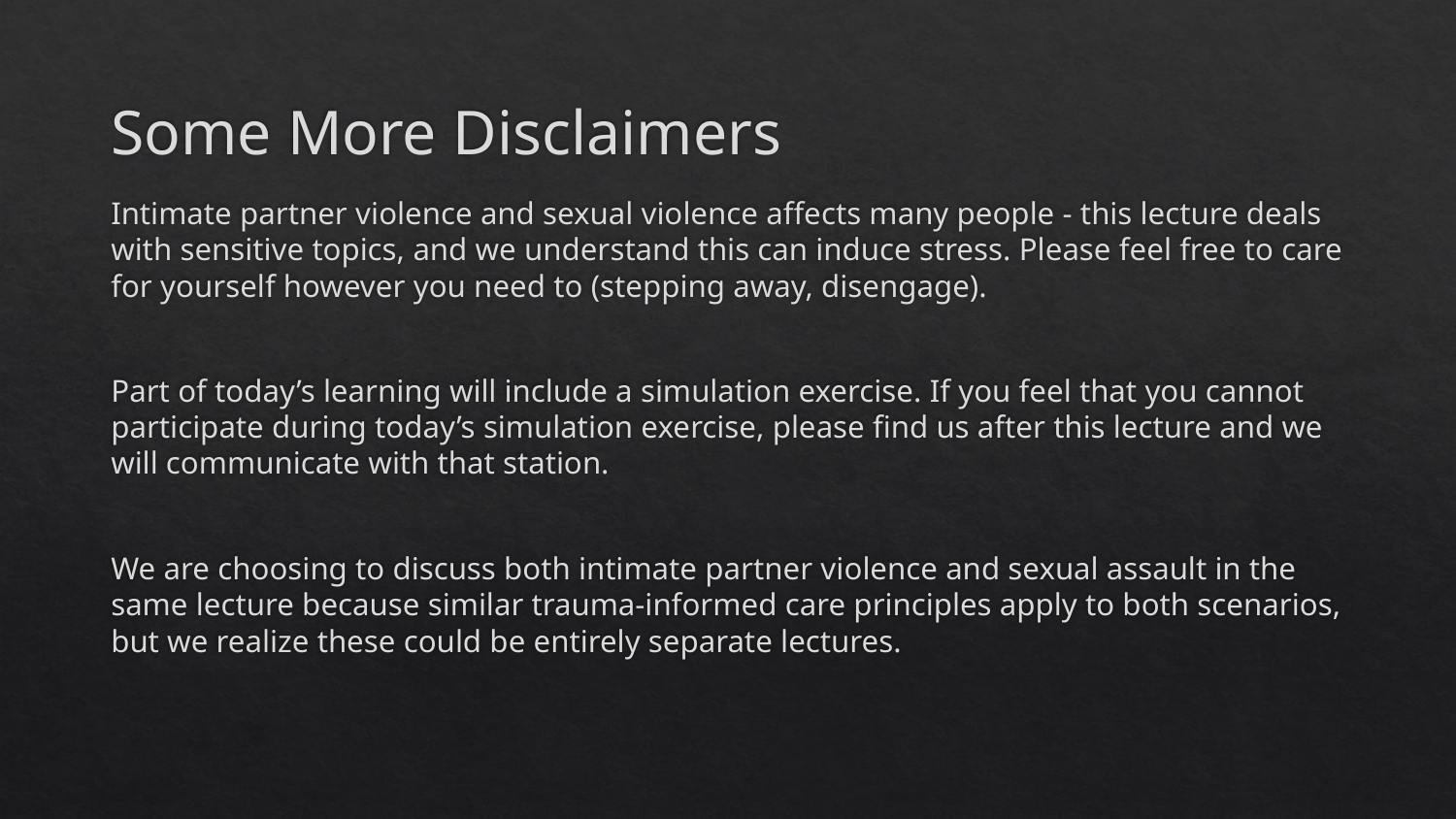

# Some More Disclaimers
Intimate partner violence and sexual violence affects many people - this lecture deals with sensitive topics, and we understand this can induce stress. Please feel free to care for yourself however you need to (stepping away, disengage).
Part of today’s learning will include a simulation exercise. If you feel that you cannot participate during today’s simulation exercise, please find us after this lecture and we will communicate with that station.
We are choosing to discuss both intimate partner violence and sexual assault in the same lecture because similar trauma-informed care principles apply to both scenarios, but we realize these could be entirely separate lectures.

## Slide 5
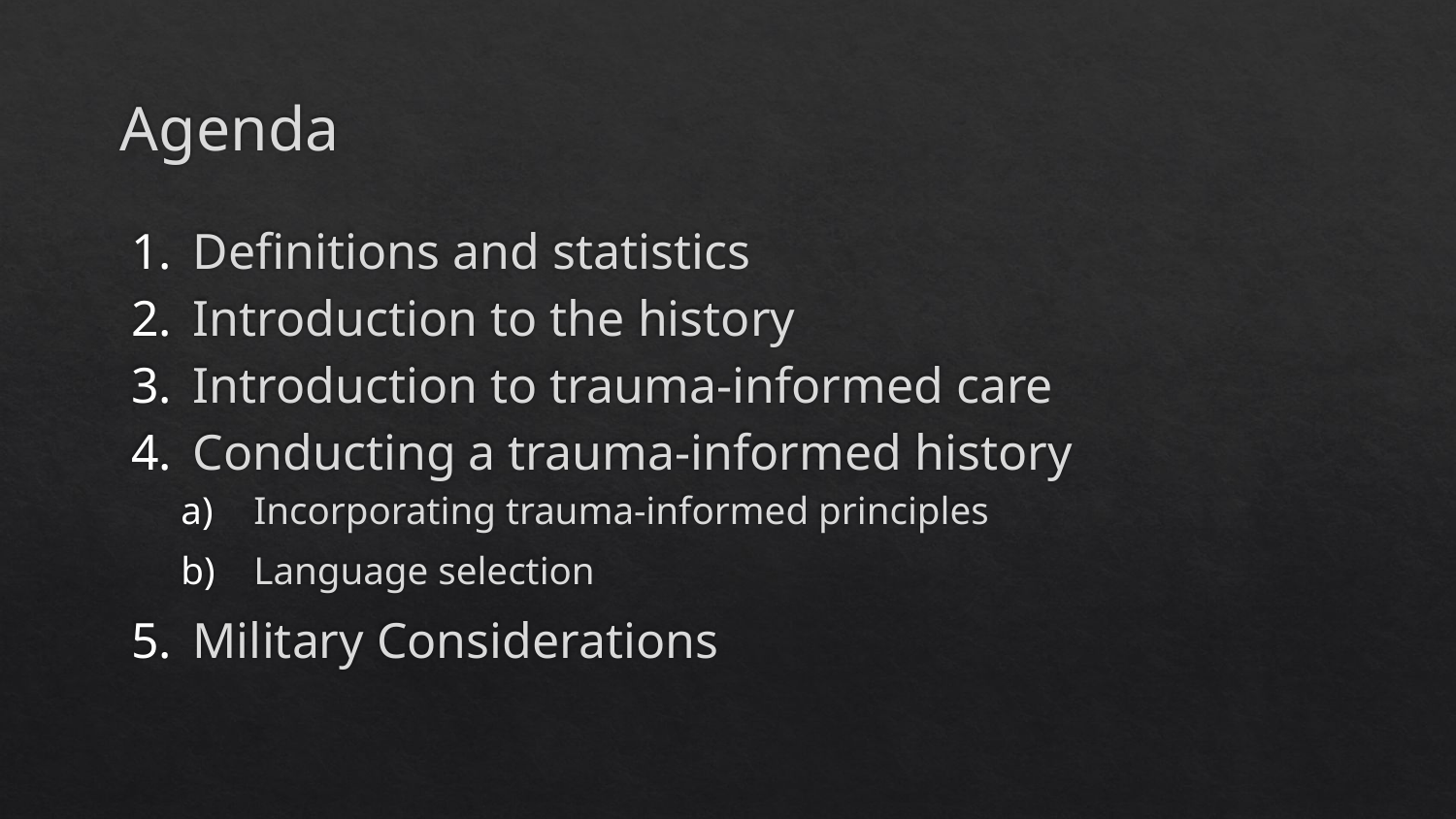

# Agenda
Definitions and statistics
Introduction to the history
Introduction to trauma-informed care
Conducting a trauma-informed history
Incorporating trauma-informed principles
Language selection
Military Considerations

## Slide 6
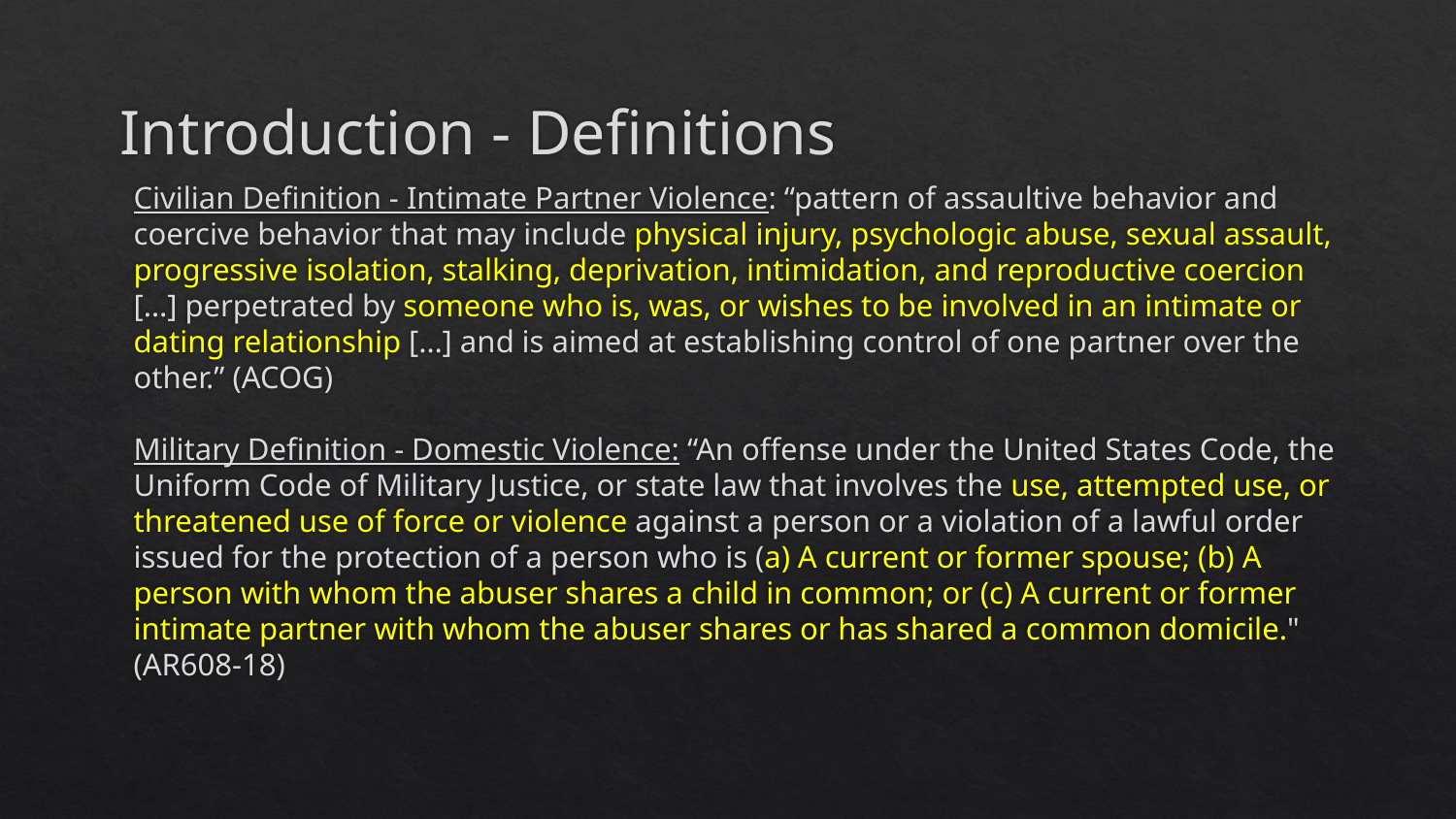

# Introduction - Definitions
Civilian Definition - Intimate Partner Violence: “pattern of assaultive behavior and coercive behavior that may include physical injury, psychologic abuse, sexual assault, progressive isolation, stalking, deprivation, intimidation, and reproductive coercion […] perpetrated by someone who is, was, or wishes to be involved in an intimate or dating relationship […] and is aimed at establishing control of one partner over the other.” (ACOG)
Military Definition - Domestic Violence: “An offense under the United States Code, the Uniform Code of Military Justice, or state law that involves the use, attempted use, or threatened use of force or violence against a person or a violation of a lawful order issued for the protection of a person who is (a) A current or former spouse; (b) A person with whom the abuser shares a child in common; or (c) A current or former intimate partner with whom the abuser shares or has shared a common domicile." (AR608-18)

## Slide 7
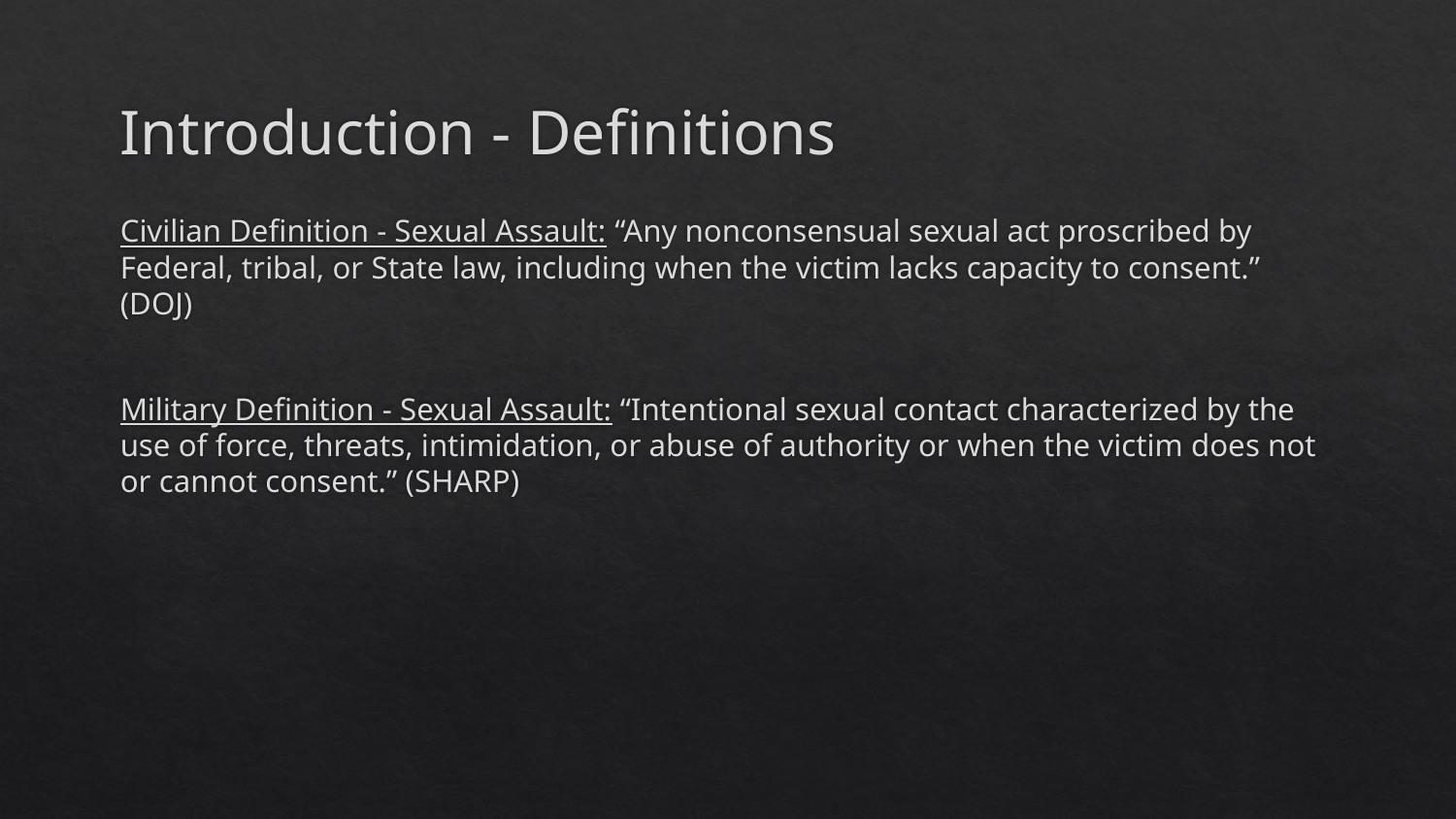

# Introduction - Definitions
Civilian Definition - Sexual Assault: “Any nonconsensual sexual act proscribed by Federal, tribal, or State law, including when the victim lacks capacity to consent.” (DOJ)
Military Definition - Sexual Assault: “Intentional sexual contact characterized by the use of force, threats, intimidation, or abuse of authority or when the victim does not or cannot consent.” (SHARP)

## Slide 8
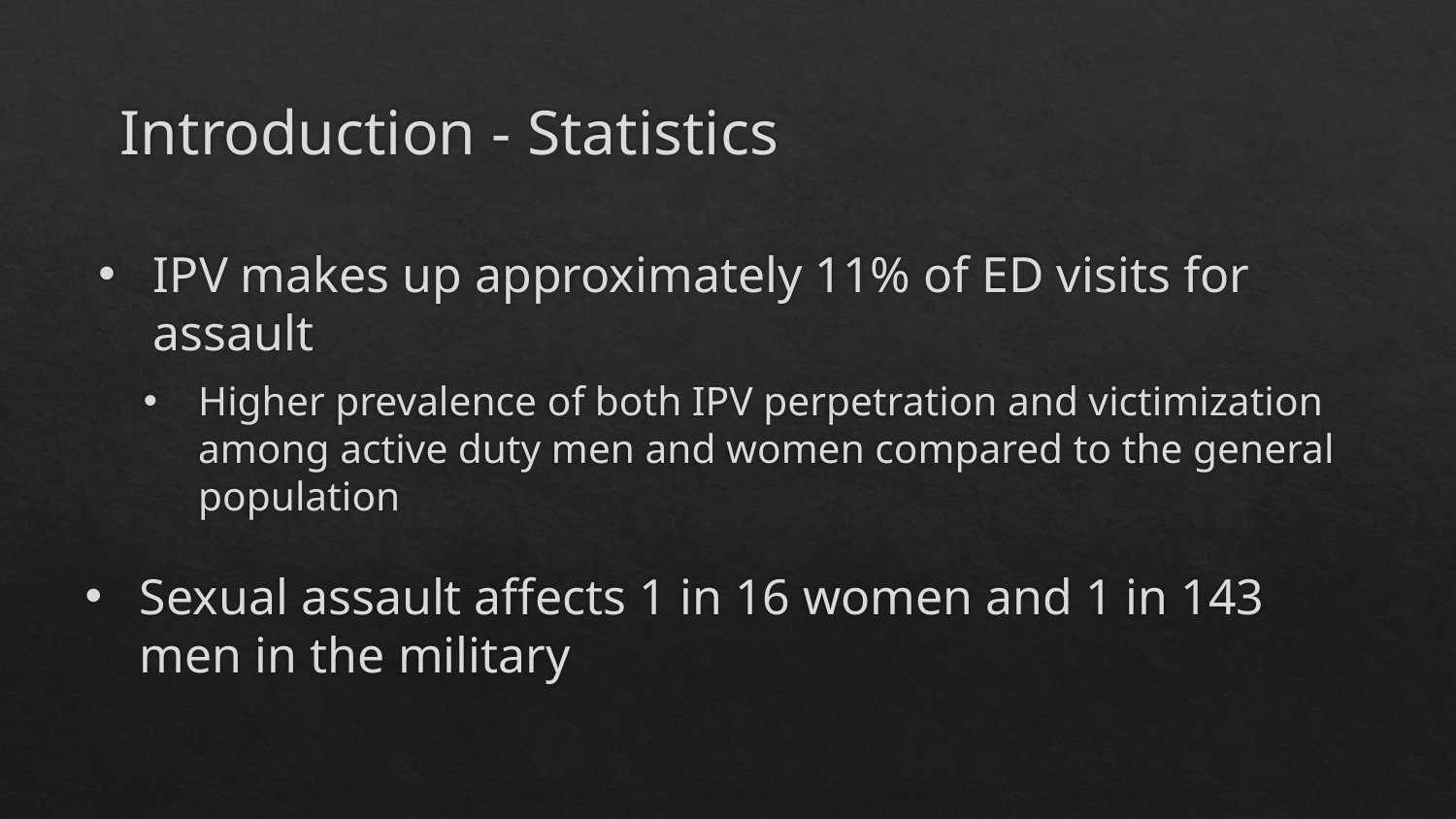

# Introduction - Statistics
IPV makes up approximately 11% of ED visits for assault
Higher prevalence of both IPV perpetration and victimization among active duty men and women compared to the general population
Sexual assault affects 1 in 16 women and 1 in 143 men in the military

## Slide 9
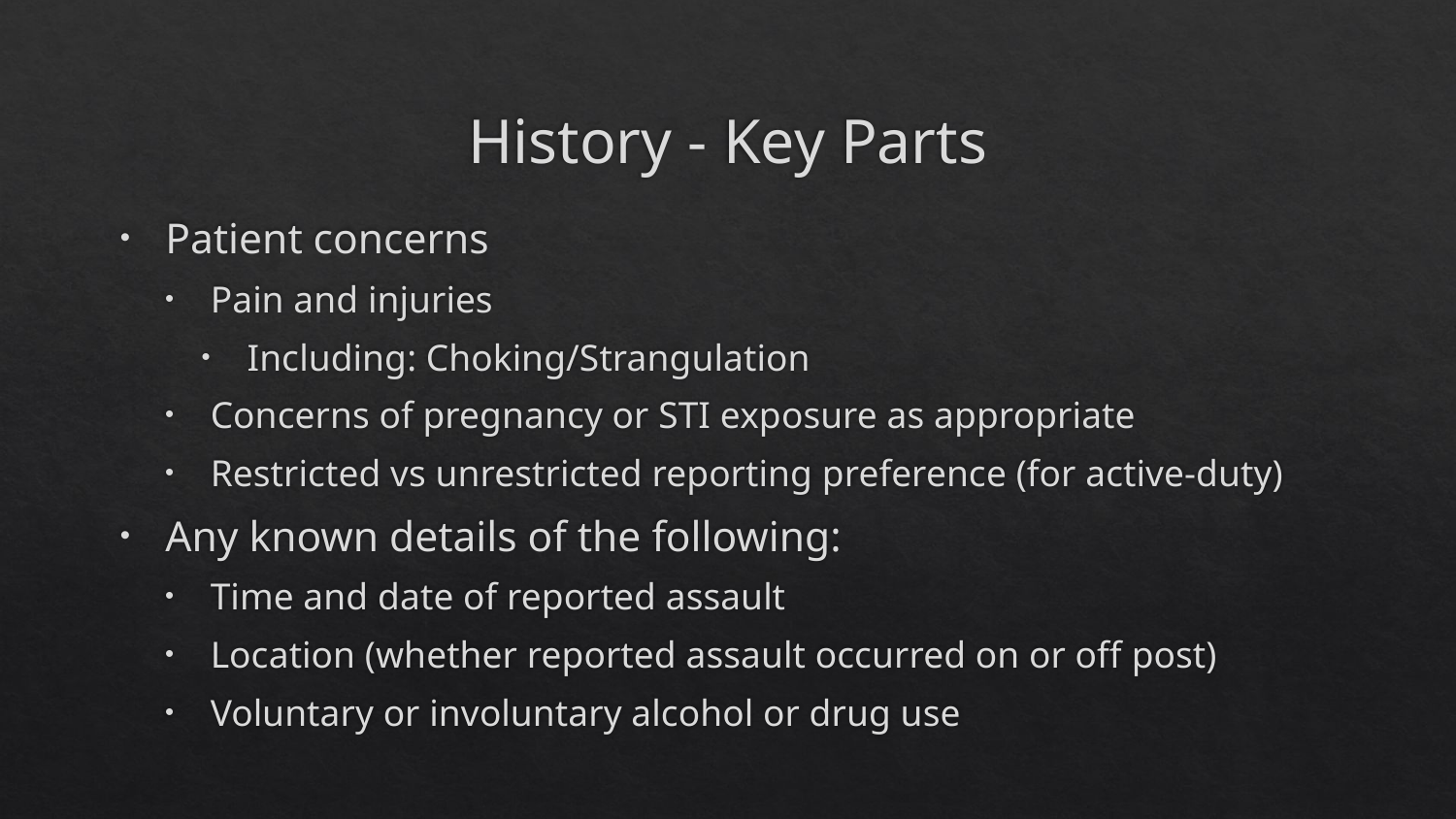

# History - Key Parts
Patient concerns
Pain and injuries
Including: Choking/Strangulation
Concerns of pregnancy or STI exposure as appropriate
Restricted vs unrestricted reporting preference (for active-duty)
Any known details of the following:
Time and date of reported assault
Location (whether reported assault occurred on or off post)
Voluntary or involuntary alcohol or drug use

## Slide 10
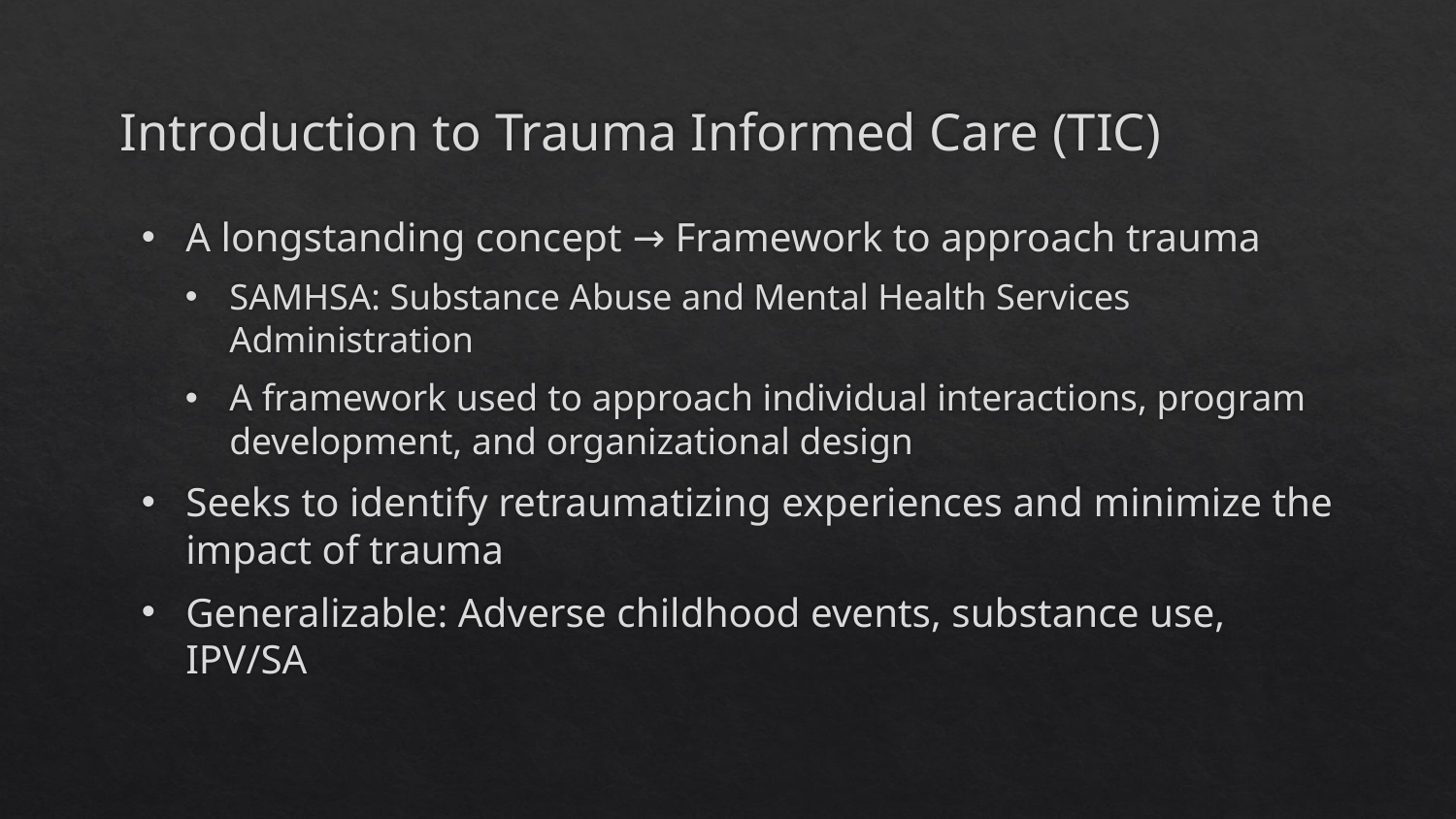

# Introduction to Trauma Informed Care (TIC)
A longstanding concept → Framework to approach trauma
SAMHSA: Substance Abuse and Mental Health Services Administration
A framework used to approach individual interactions, program development, and organizational design
Seeks to identify retraumatizing experiences and minimize the impact of trauma
Generalizable: Adverse childhood events, substance use, IPV/SA

## Slide 11
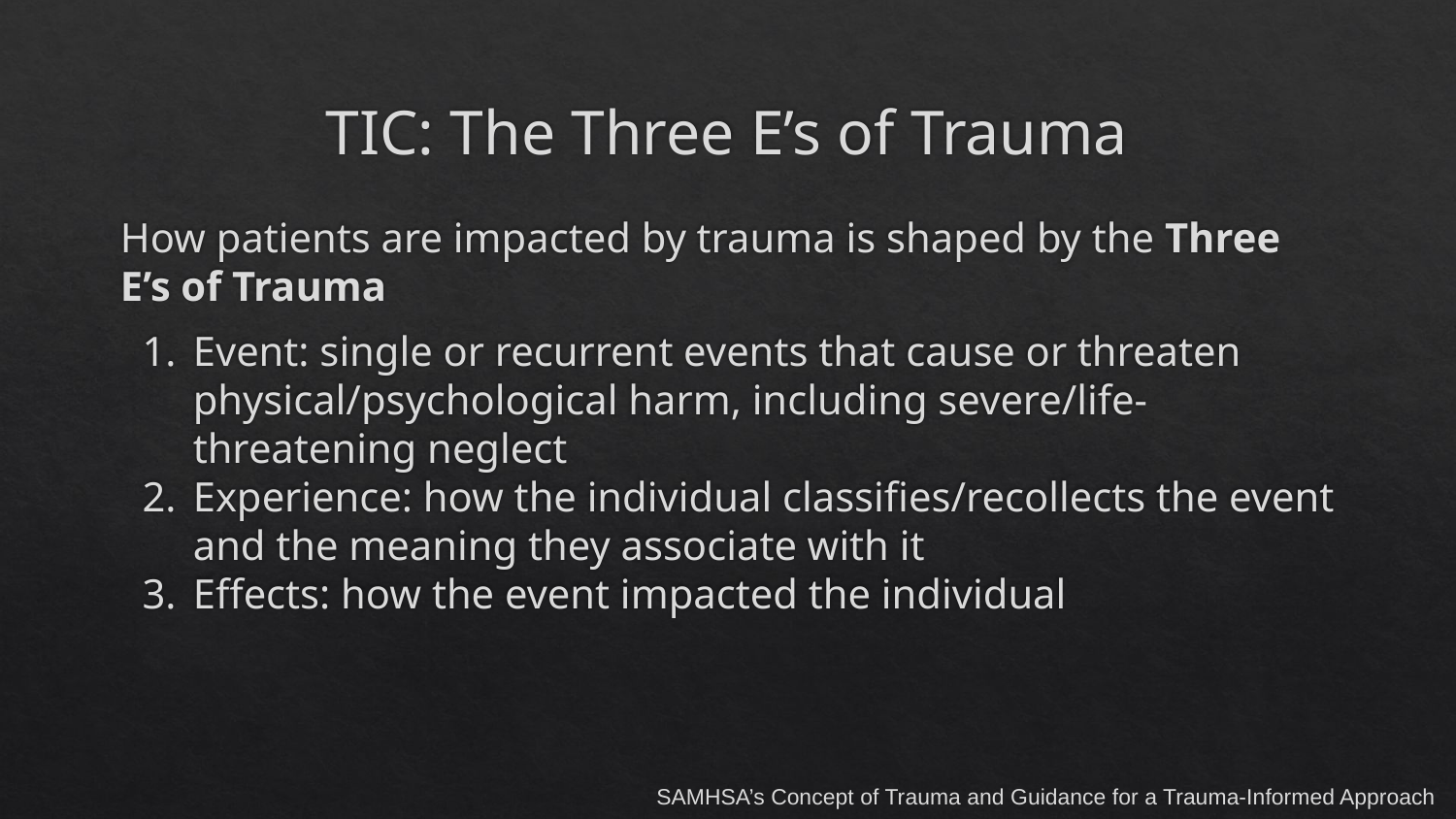

# TIC: The Three E’s of Trauma
How patients are impacted by trauma is shaped by the Three E’s of Trauma
Event: single or recurrent events that cause or threaten physical/psychological harm, including severe/life-threatening neglect
Experience: how the individual classifies/recollects the event and the meaning they associate with it
Effects: how the event impacted the individual
SAMHSA’s Concept of Trauma and Guidance for a Trauma-Informed Approach

## Slide 12
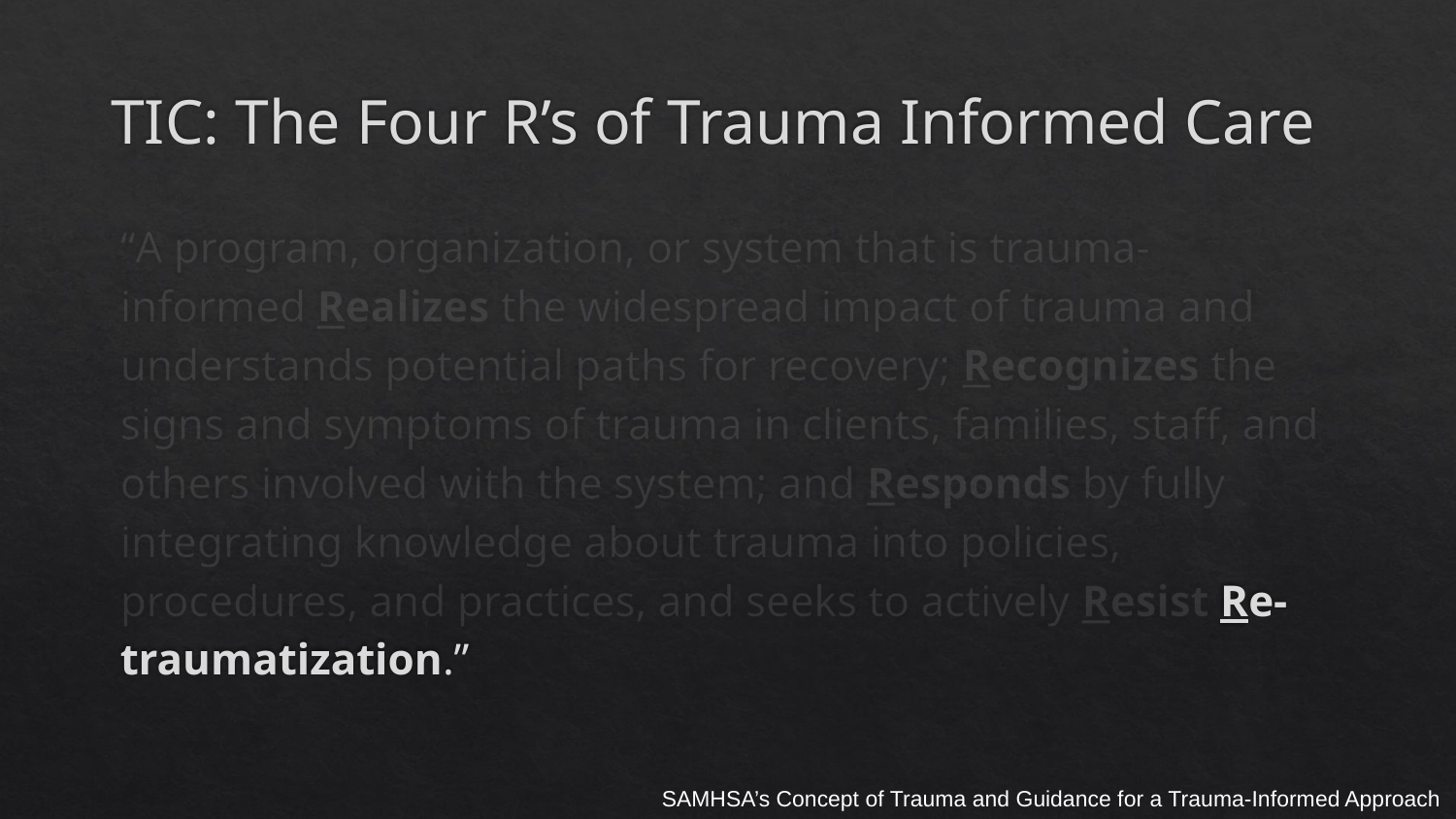

# TIC: The Four R’s of Trauma Informed Care
“A program, organization, or system that is trauma-informed Realizes the widespread impact of trauma and understands potential paths for recovery; Recognizes the signs and symptoms of trauma in clients, families, staff, and others involved with the system; and Responds by fully integrating knowledge about trauma into policies, procedures, and practices, and seeks to actively Resist Re-traumatization.”
SAMHSA’s Concept of Trauma and Guidance for a Trauma-Informed Approach

## Slide 13
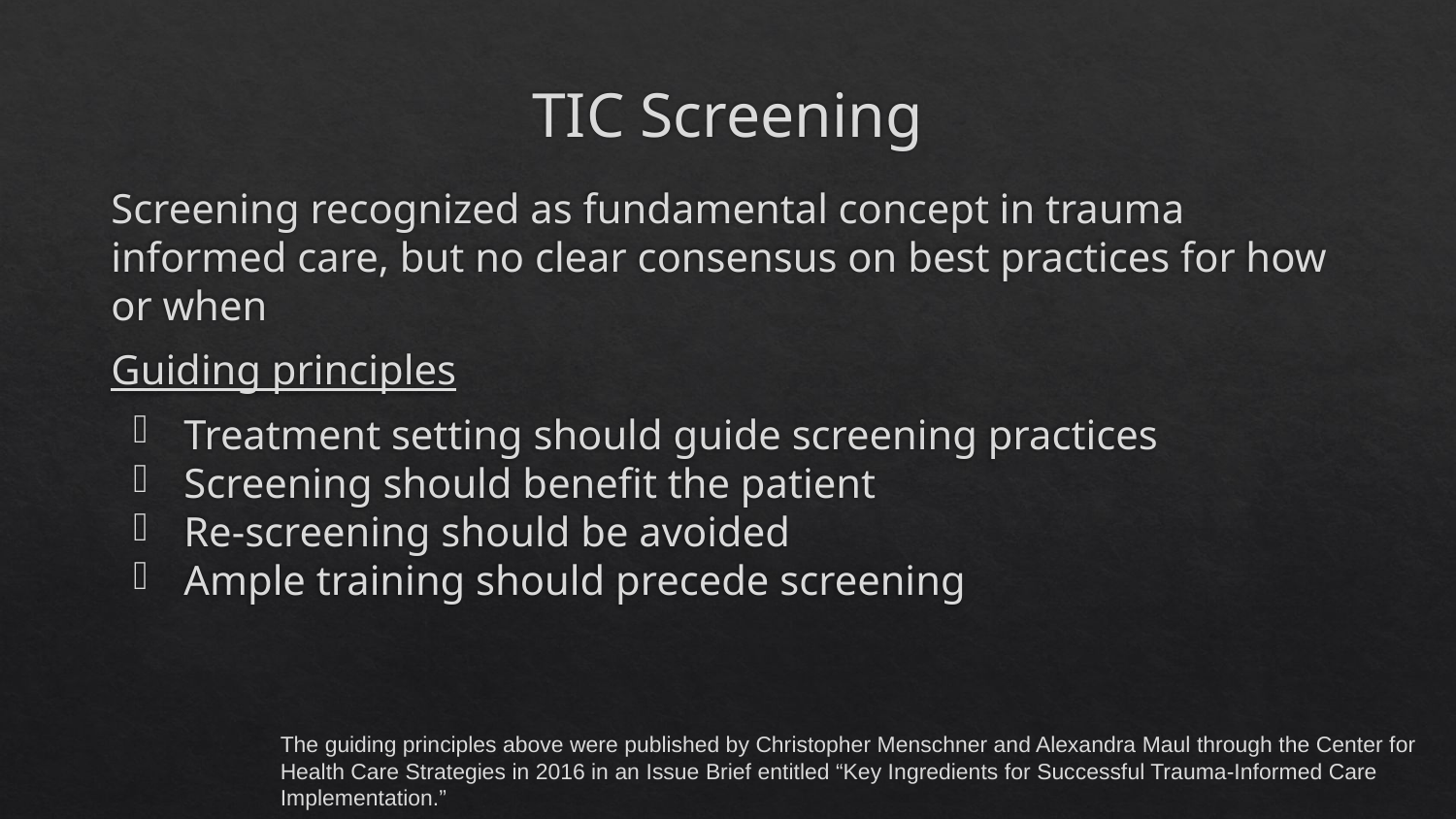

# TIC Screening
Screening recognized as fundamental concept in trauma informed care, but no clear consensus on best practices for how or when
Guiding principles
Treatment setting should guide screening practices
Screening should benefit the patient
Re-screening should be avoided
Ample training should precede screening
The guiding principles above were published by Christopher Menschner and Alexandra Maul through the Center for Health Care Strategies in 2016 in an Issue Brief entitled “Key Ingredients for Successful Trauma-Informed Care Implementation.”

## Slide 14
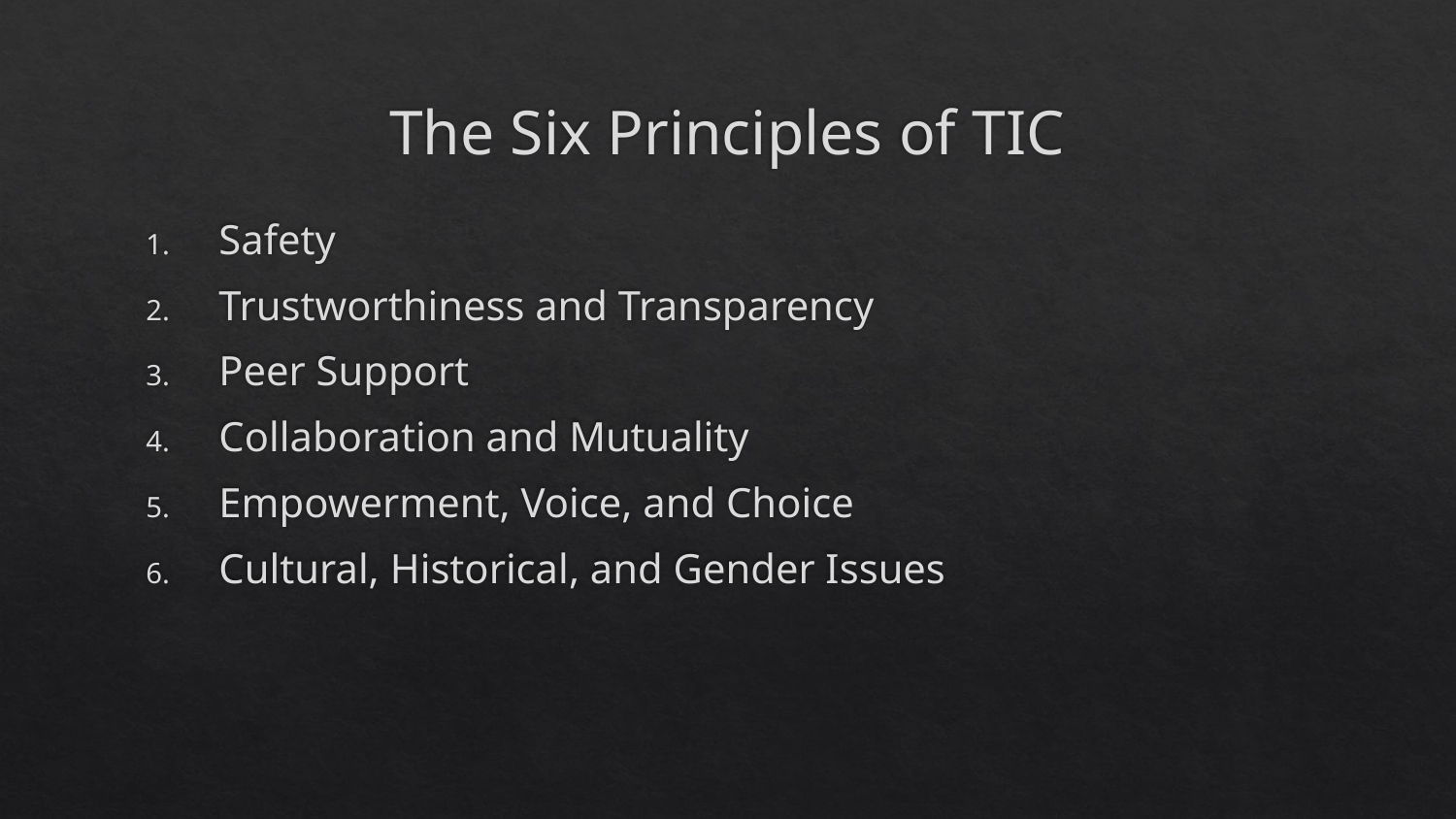

# The Six Principles of TIC
Safety
Trustworthiness and Transparency
Peer Support
Collaboration and Mutuality
Empowerment, Voice, and Choice
Cultural, Historical, and Gender Issues

## Slide 15
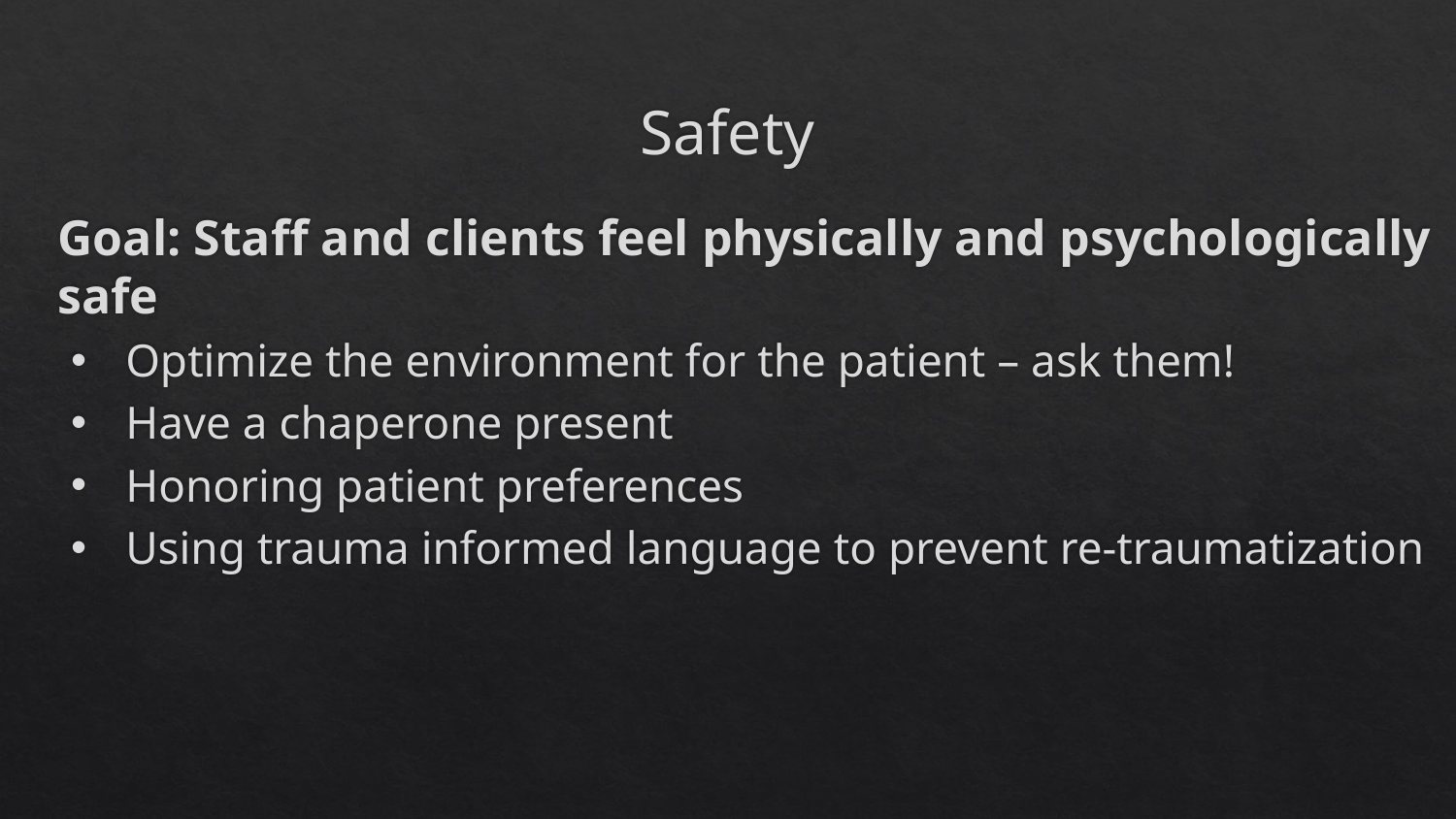

# Safety
Goal: Staff and clients feel physically and psychologically safe
Optimize the environment for the patient – ask them!
Have a chaperone present
Honoring patient preferences
Using trauma informed language to prevent re-traumatization

## Slide 16
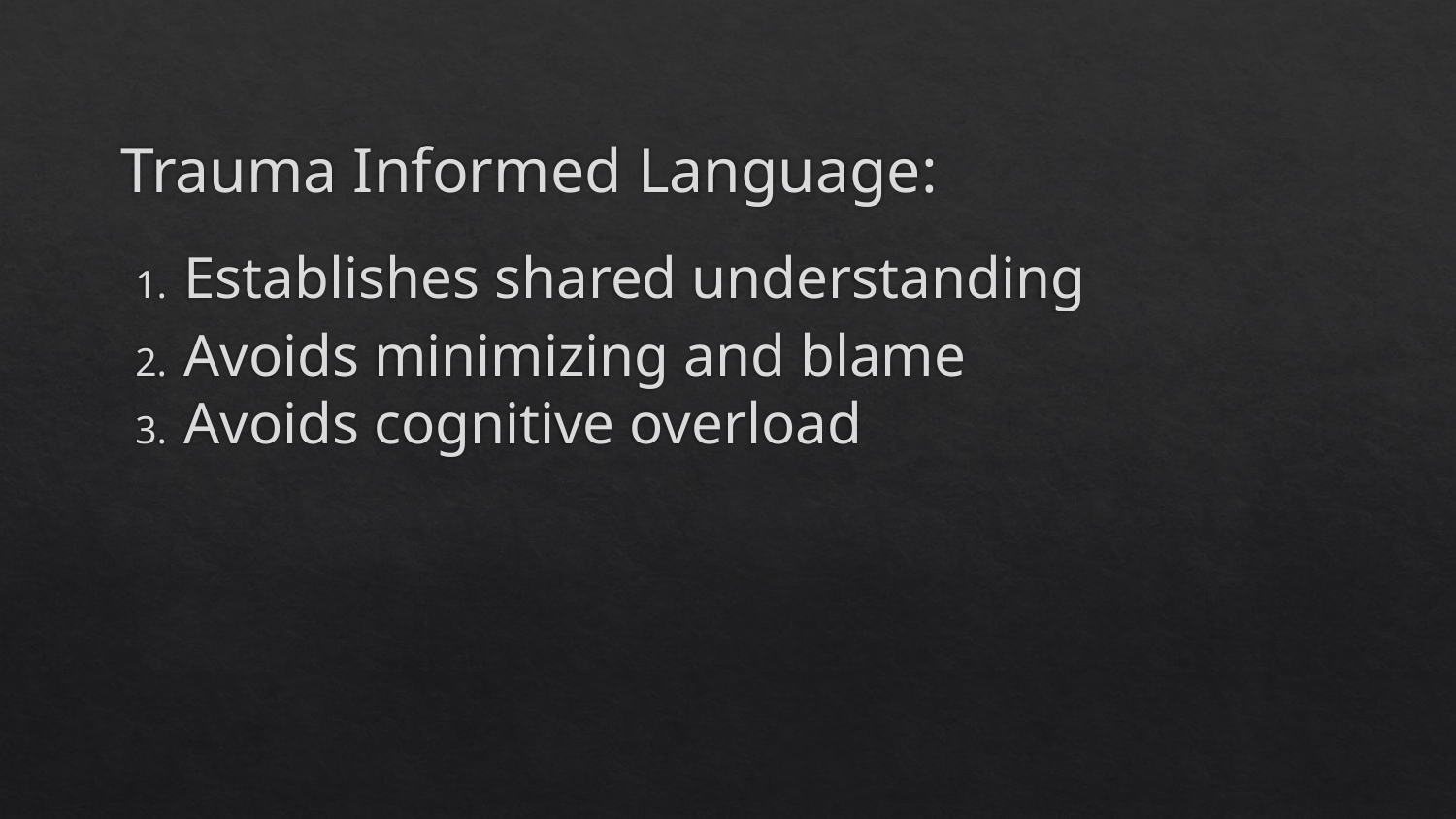

# Trauma Informed Language:
Establishes shared understanding
Avoids minimizing and blame
Avoids cognitive overload

## Slide 17
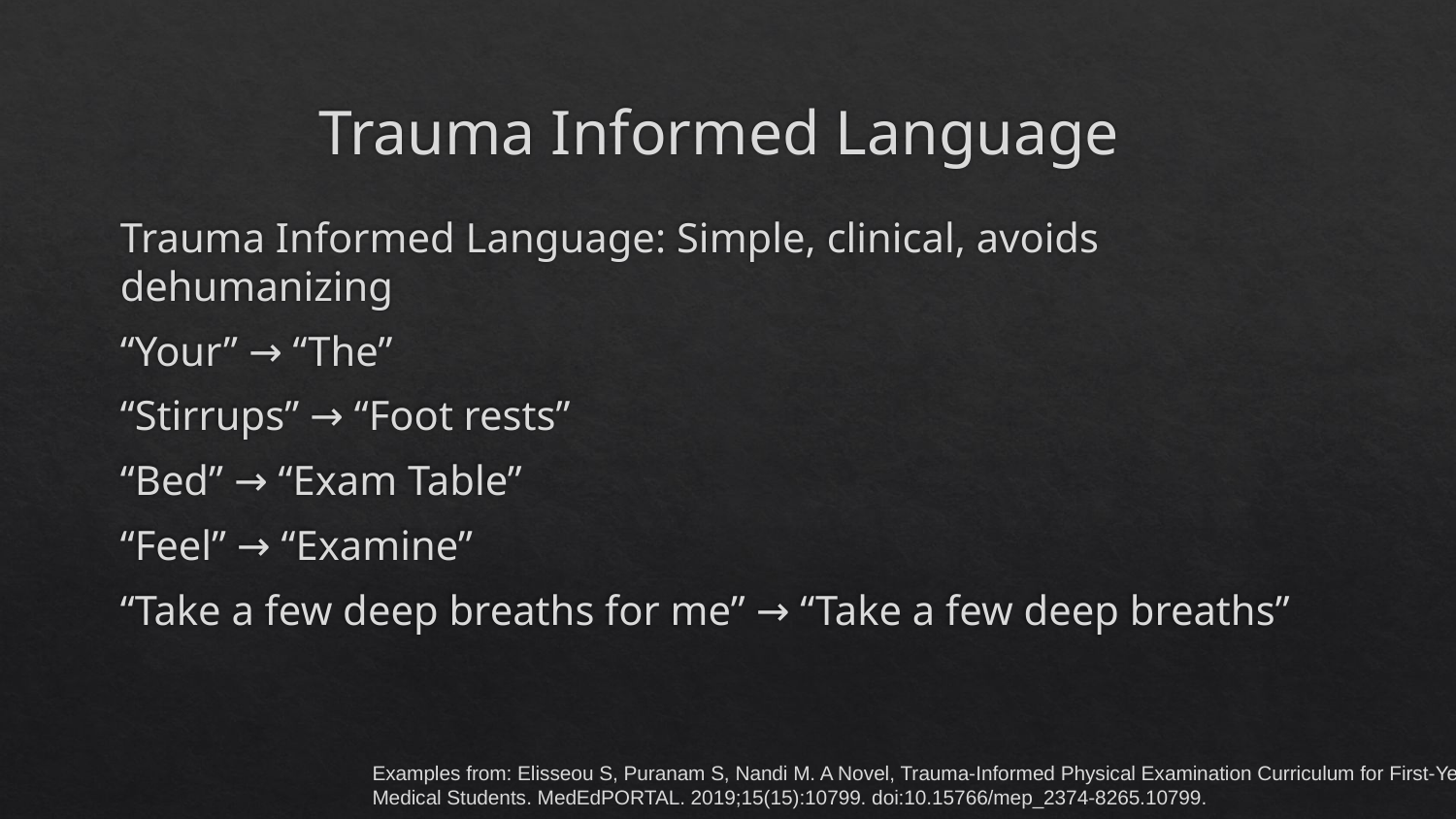

# Trauma Informed Language
Trauma Informed Language: Simple, clinical, avoids dehumanizing
“Your” → “The”
“Stirrups” → “Foot rests”
“Bed” → “Exam Table”
“Feel” → “Examine”
“Take a few deep breaths for me” → “Take a few deep breaths”
Examples from: Elisseou S, Puranam S, Nandi M. A Novel, Trauma-Informed Physical Examination Curriculum for First-Year Medical Students. MedEdPORTAL. 2019;15(15):10799. doi:10.15766/mep_2374-8265.10799.

## Slide 18
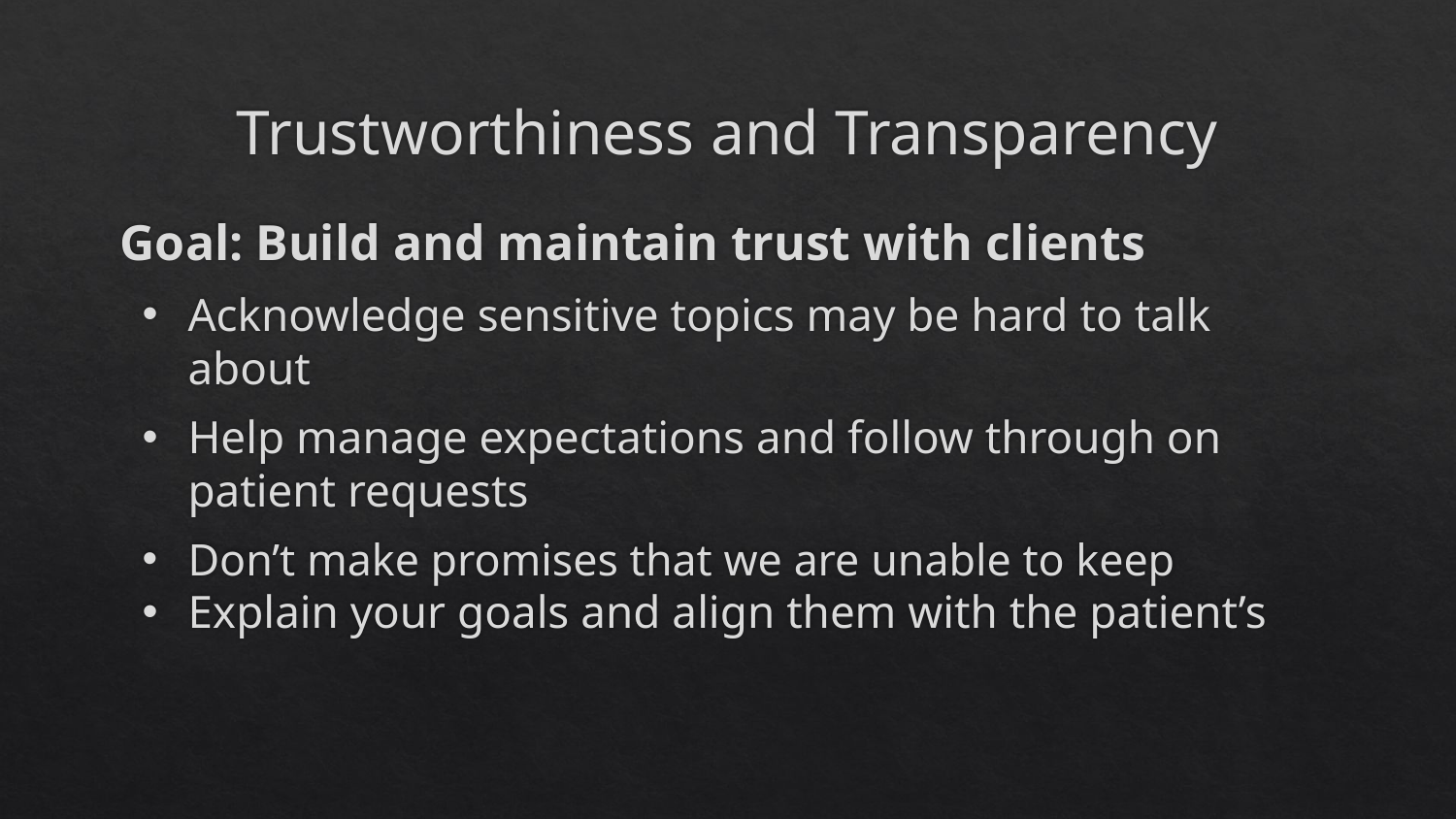

# Trustworthiness and Transparency
Goal: Build and maintain trust with clients
Acknowledge sensitive topics may be hard to talk about
Help manage expectations and follow through on patient requests
Don’t make promises that we are unable to keep
Explain your goals and align them with the patient’s

## Slide 19
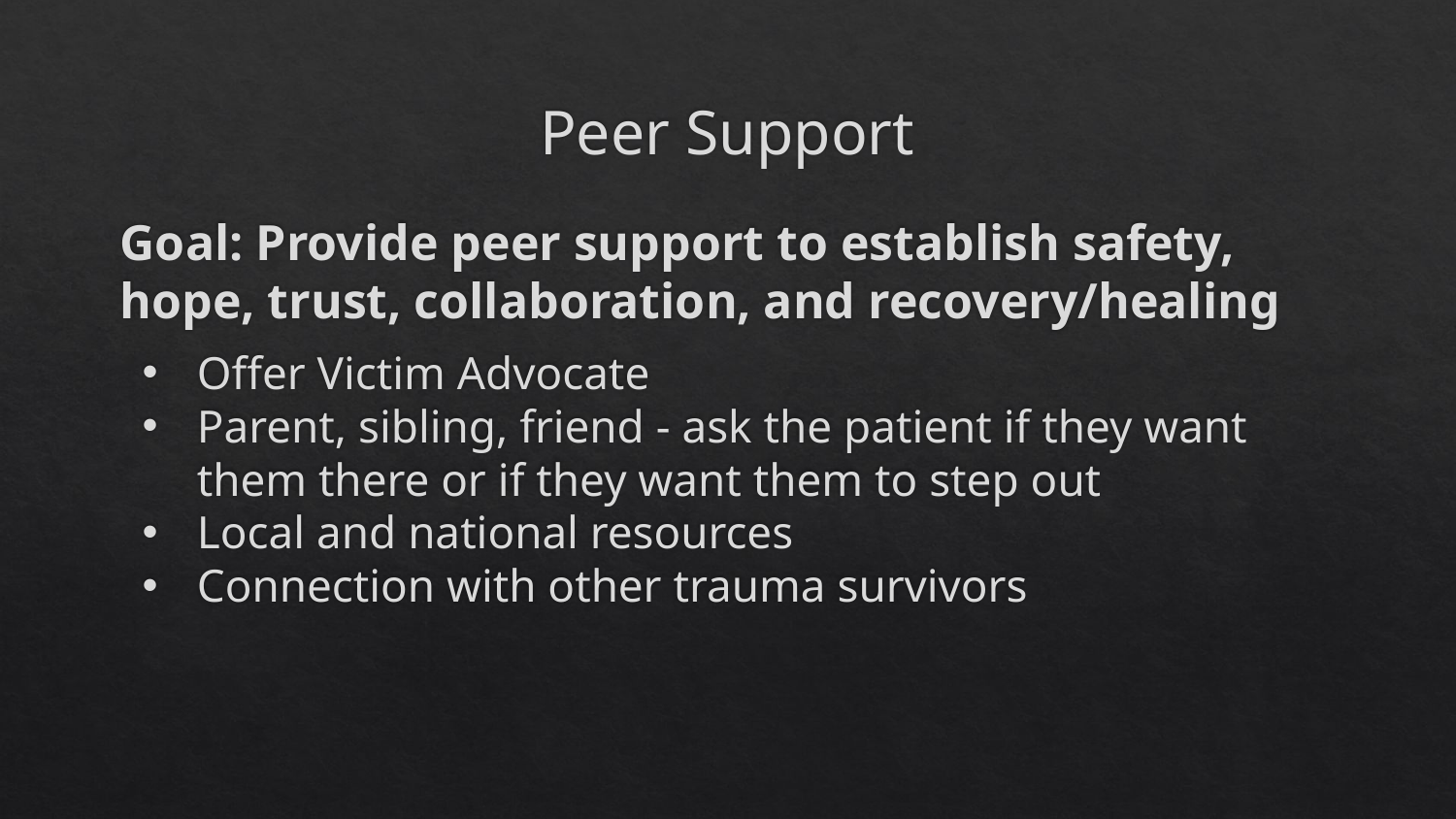

# Peer Support
Goal: Provide peer support to establish safety, hope, trust, collaboration, and recovery/healing
Offer Victim Advocate
Parent, sibling, friend - ask the patient if they want them there or if they want them to step out
Local and national resources
Connection with other trauma survivors

## Slide 20
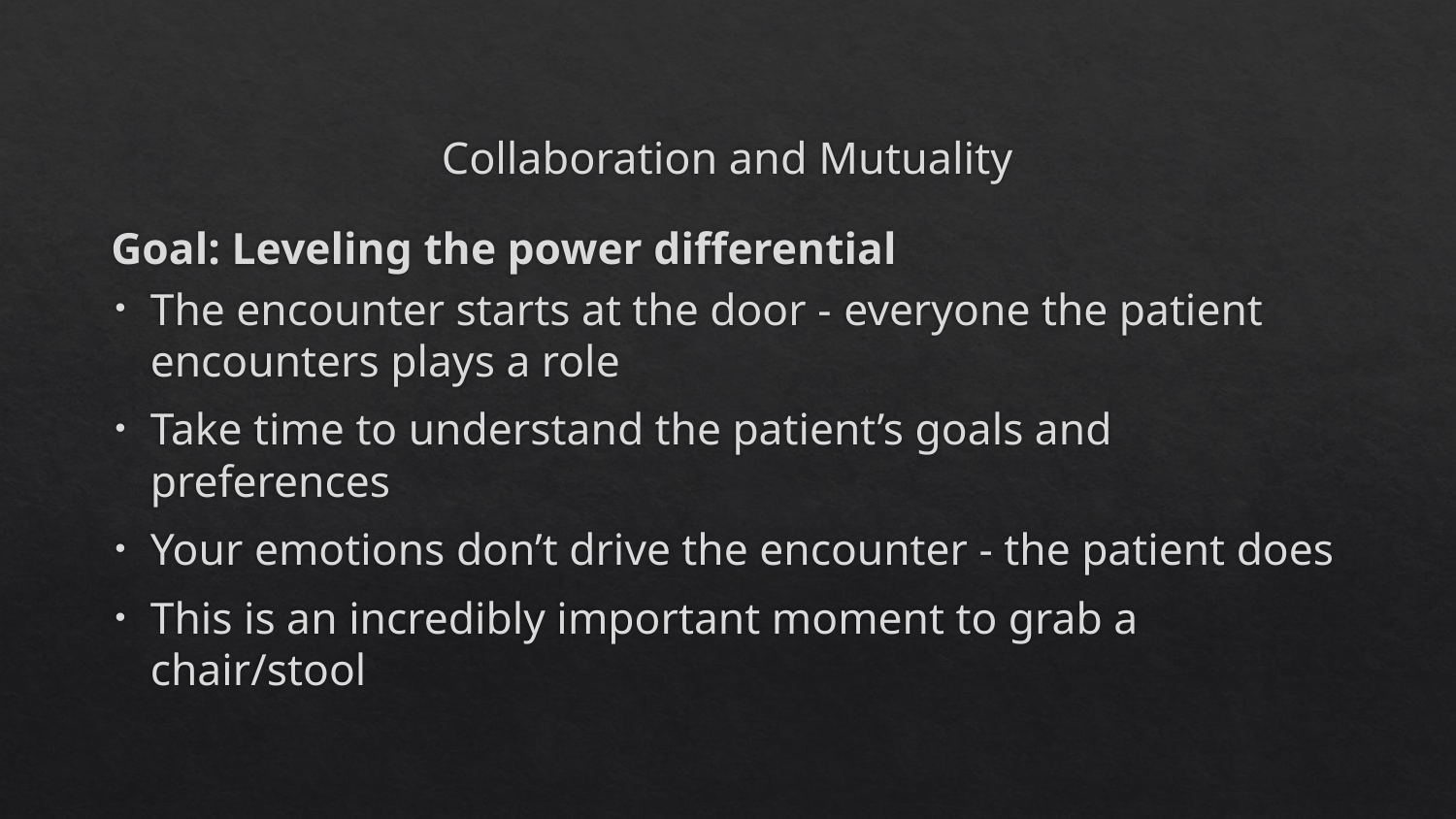

# Collaboration and Mutuality
Goal: Leveling the power differential
The encounter starts at the door - everyone the patient encounters plays a role
Take time to understand the patient’s goals and preferences
Your emotions don’t drive the encounter - the patient does
This is an incredibly important moment to grab a chair/stool

## Slide 21
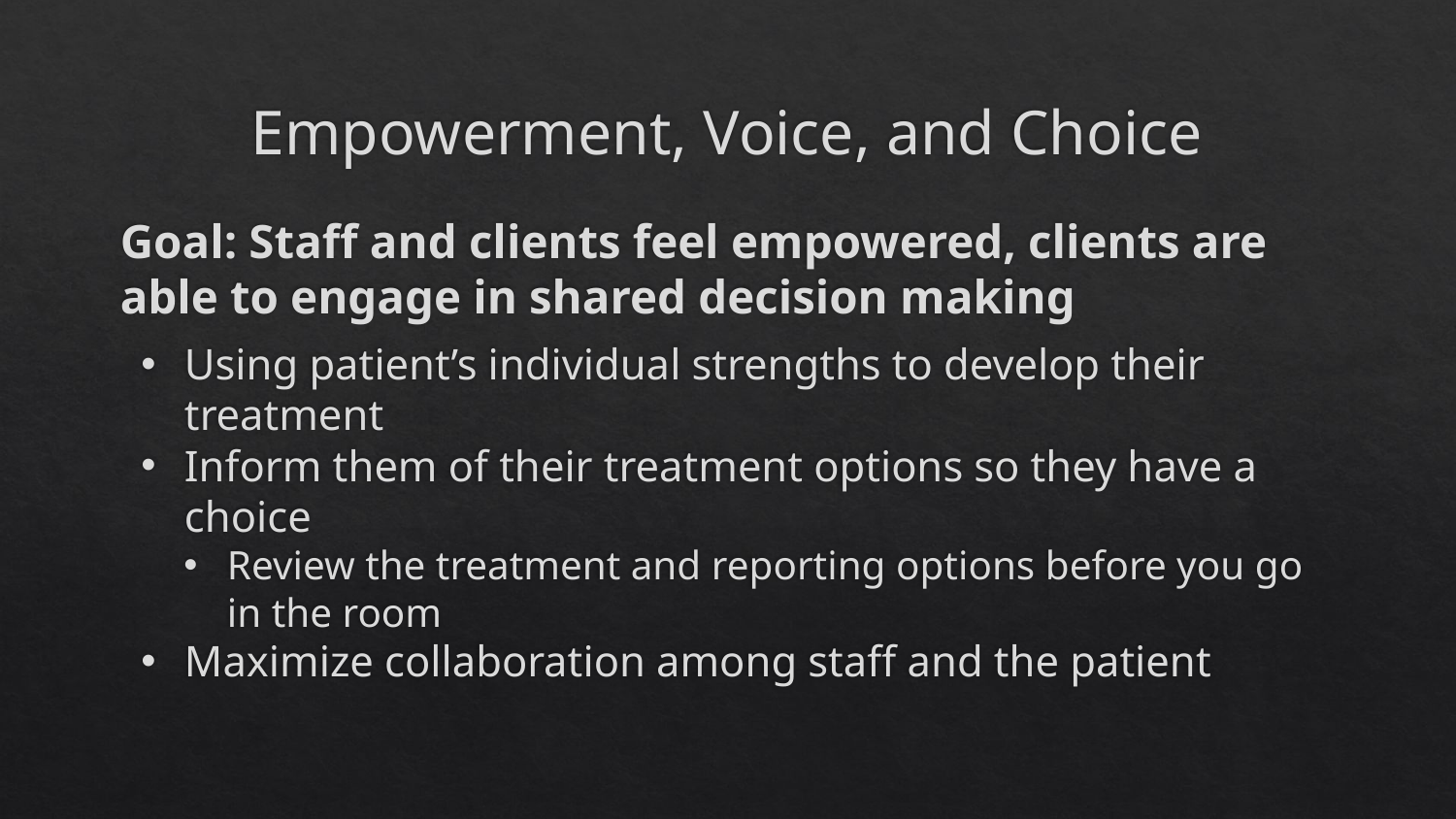

# Empowerment, Voice, and Choice
Goal: Staff and clients feel empowered, clients are able to engage in shared decision making
Using patient’s individual strengths to develop their treatment
Inform them of their treatment options so they have a choice
Review the treatment and reporting options before you go in the room
Maximize collaboration among staff and the patient

## Slide 22
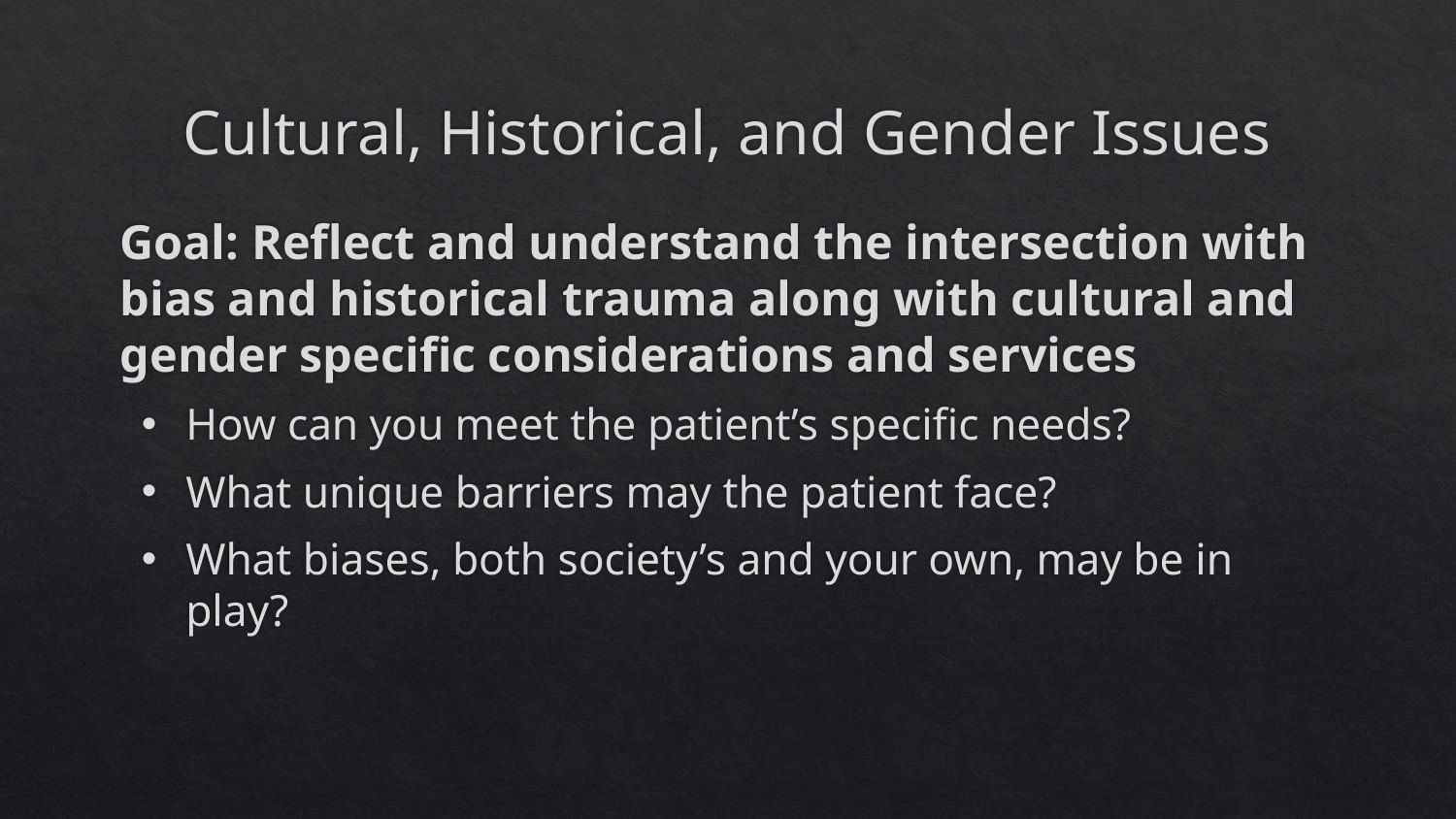

# Cultural, Historical, and Gender Issues
Goal: Reflect and understand the intersection with bias and historical trauma along with cultural and gender specific considerations and services
How can you meet the patient’s specific needs?
What unique barriers may the patient face?
What biases, both society’s and your own, may be in play?

## Slide 23
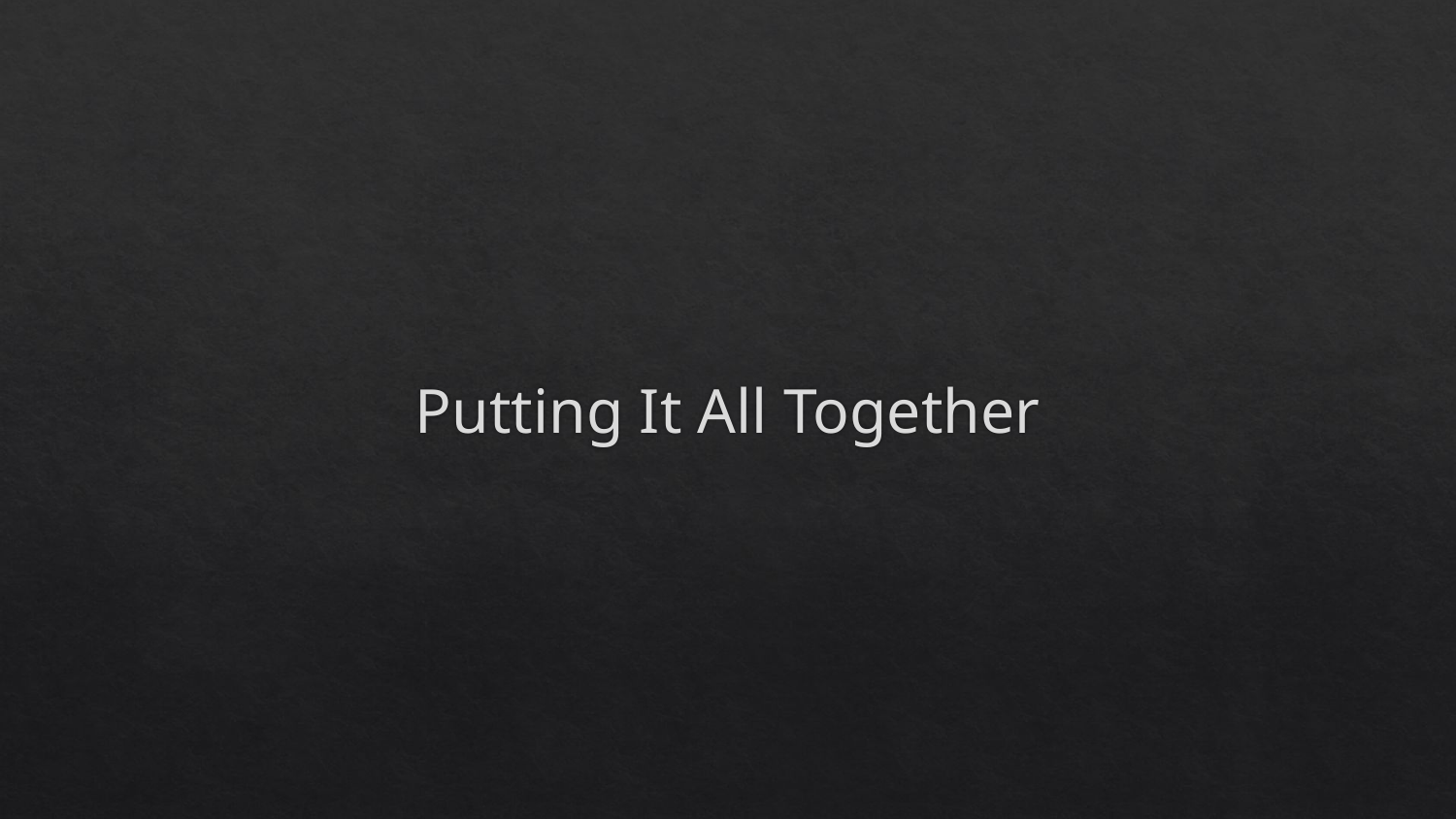

# Putting It All Together

## Slide 24
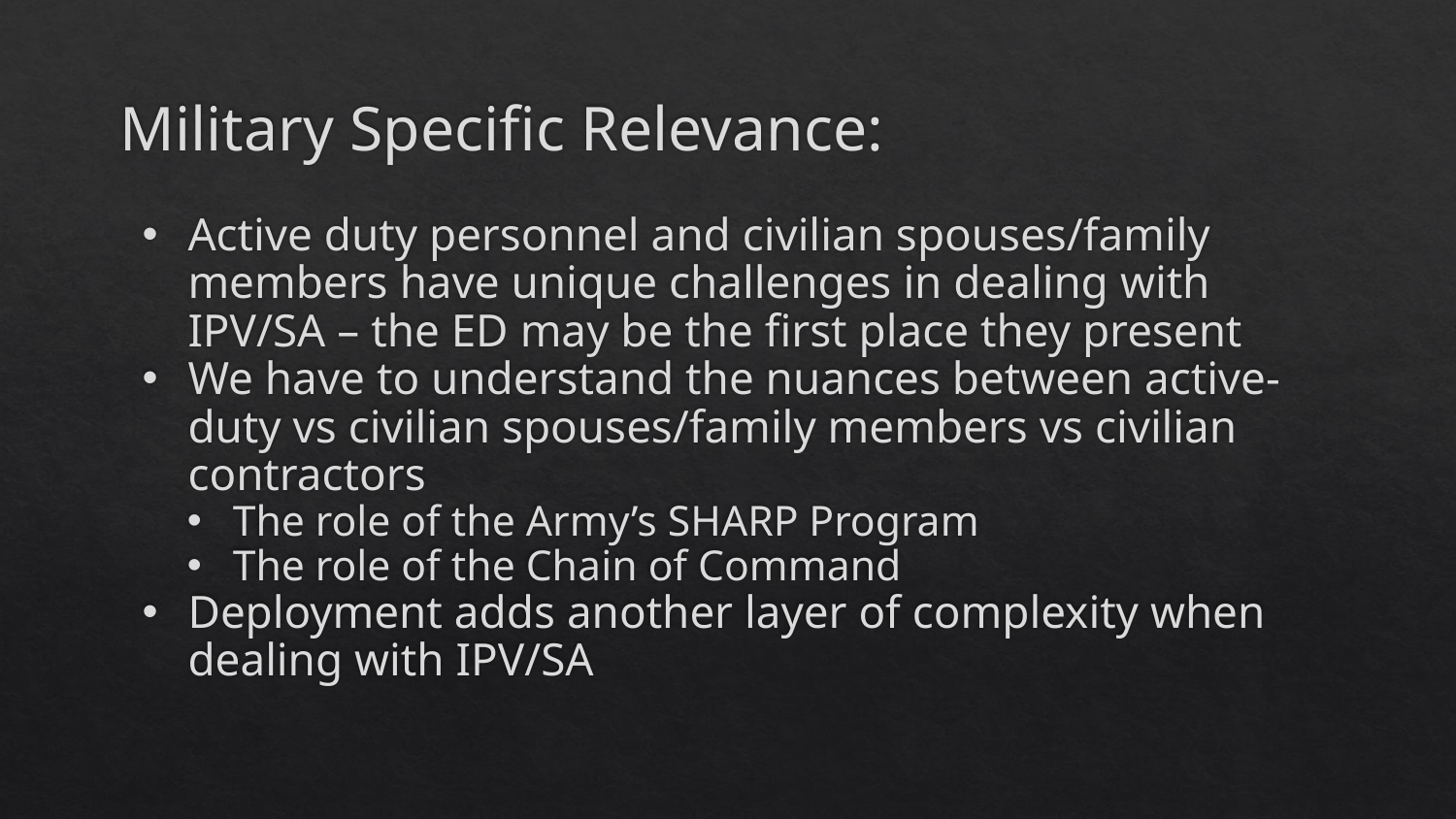

# Military Specific Relevance:
Active duty personnel and civilian spouses/family members have unique challenges in dealing with IPV/SA – the ED may be the first place they present
We have to understand the nuances between active-duty vs civilian spouses/family members vs civilian contractors
The role of the Army’s SHARP Program
The role of the Chain of Command
Deployment adds another layer of complexity when dealing with IPV/SA

## Slide 25
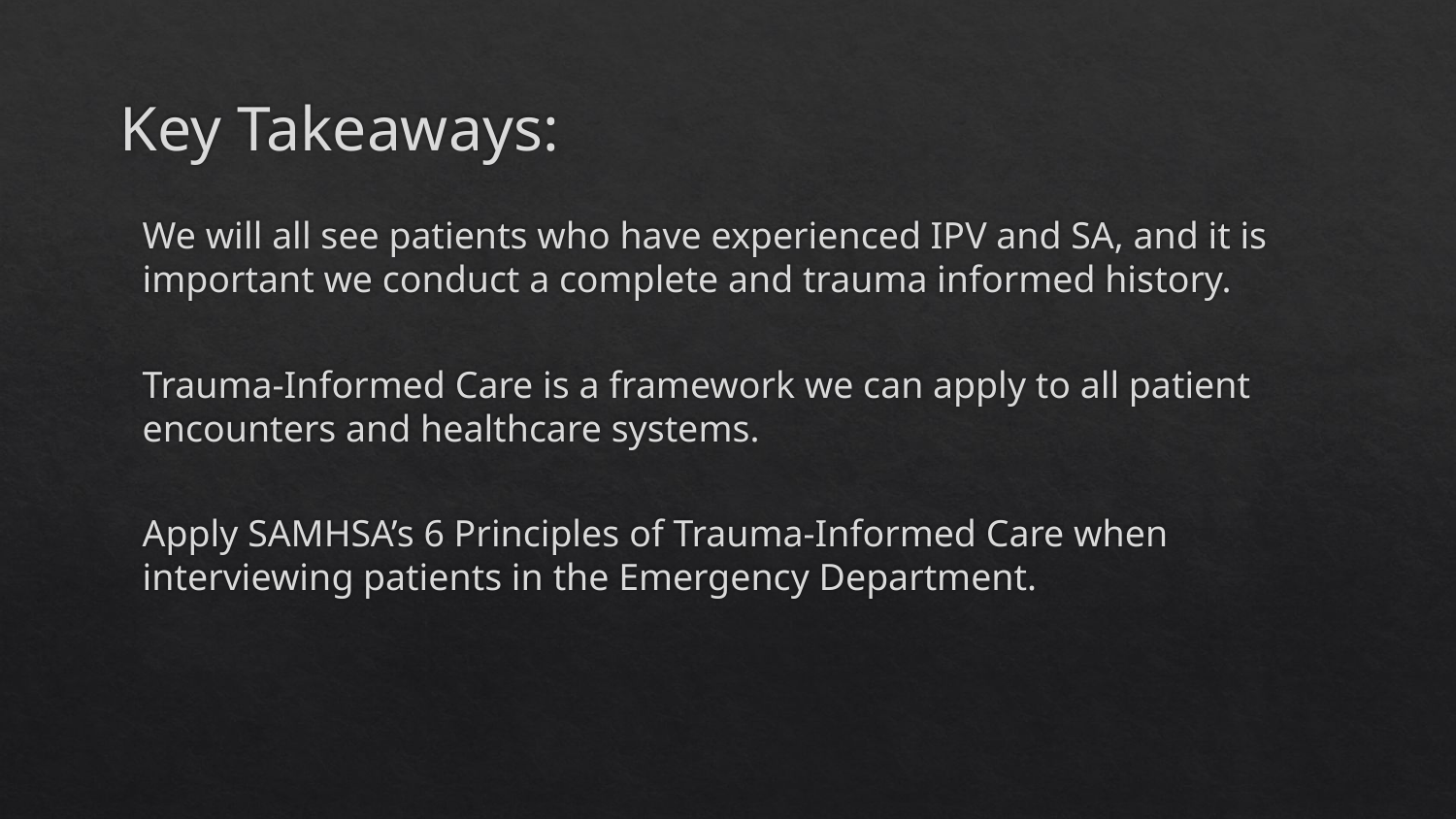

# Key Takeaways:
We will all see patients who have experienced IPV and SA, and it is important we conduct a complete and trauma informed history.
Trauma-Informed Care is a framework we can apply to all patient encounters and healthcare systems.
Apply SAMHSA’s 6 Principles of Trauma-Informed Care when interviewing patients in the Emergency Department.

## Slide 26
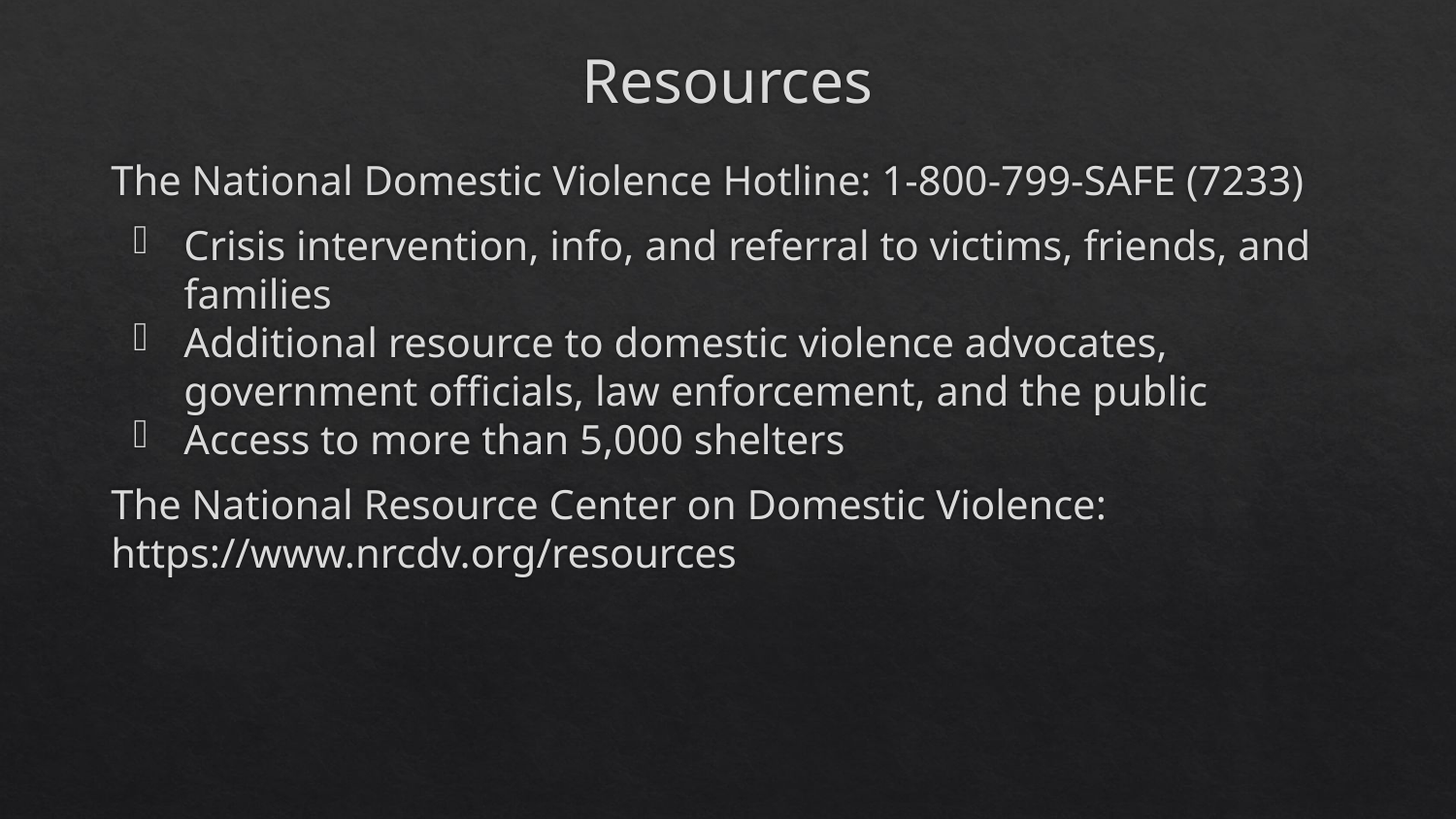

# Resources
The National Domestic Violence Hotline: 1-800-799-SAFE (7233)
Crisis intervention, info, and referral to victims, friends, and families
Additional resource to domestic violence advocates, government officials, law enforcement, and the public
Access to more than 5,000 shelters
The National Resource Center on Domestic Violence: https://www.nrcdv.org/resources

## Slide 27
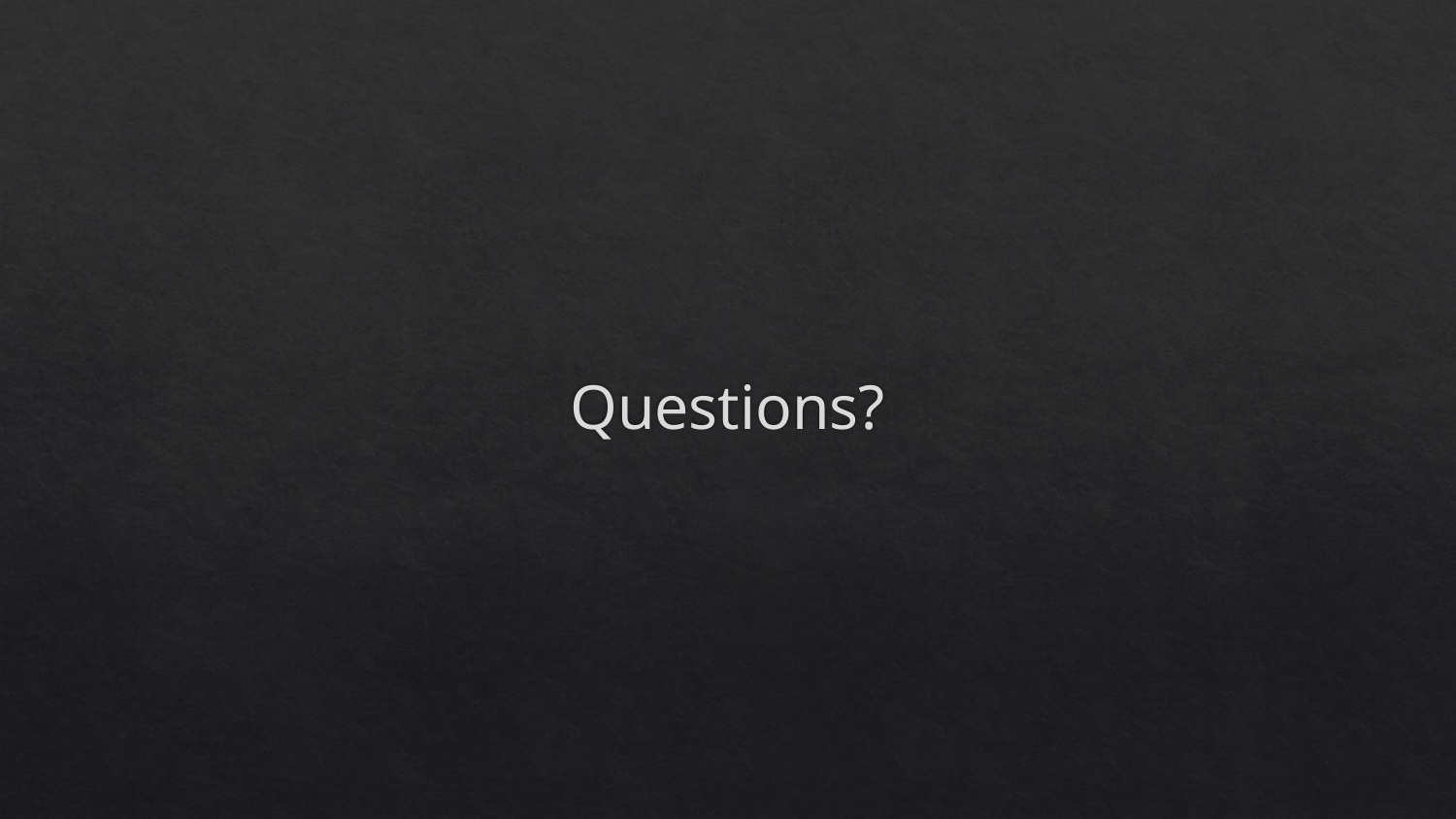

# Questions?

## Slide 28
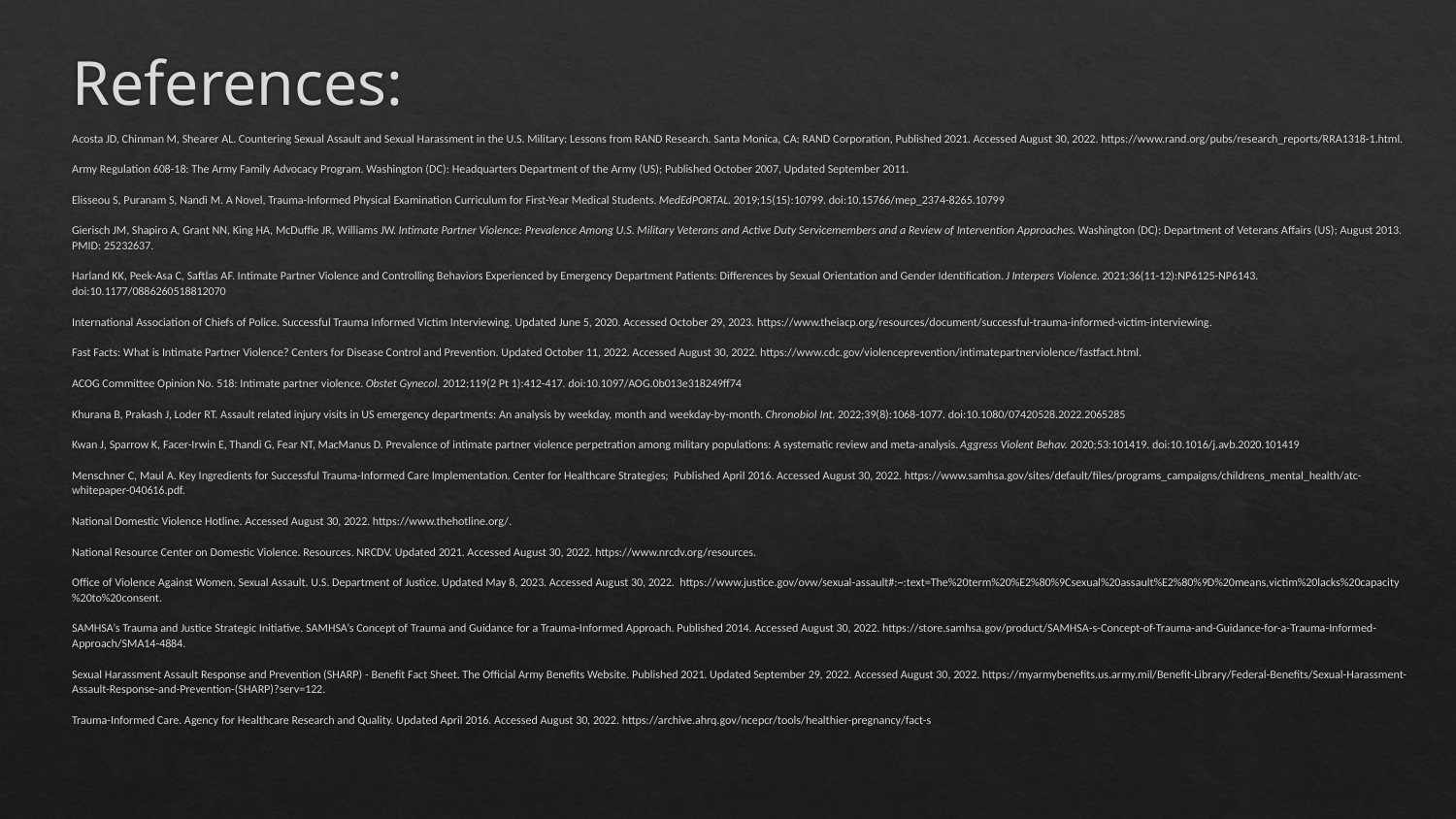

# References:
Acosta JD, Chinman M, Shearer AL. Countering Sexual Assault and Sexual Harassment in the U.S. Military: Lessons from RAND Research. Santa Monica, CA: RAND Corporation, Published 2021. Accessed August 30, 2022. https://www.rand.org/pubs/research_reports/RRA1318-1.html.
Army Regulation 608-18: The Army Family Advocacy Program. Washington (DC): Headquarters Department of the Army (US); Published October 2007, Updated September 2011.
Elisseou S, Puranam S, Nandi M. A Novel, Trauma-Informed Physical Examination Curriculum for First-Year Medical Students. MedEdPORTAL. 2019;15(15):10799. doi:10.15766/mep_2374-8265.10799
Gierisch JM, Shapiro A, Grant NN, King HA, McDuffie JR, Williams JW. Intimate Partner Violence: Prevalence Among U.S. Military Veterans and Active Duty Servicemembers and a Review of Intervention Approaches. Washington (DC): Department of Veterans Affairs (US); August 2013. PMID: 25232637.
Harland KK, Peek-Asa C, Saftlas AF. Intimate Partner Violence and Controlling Behaviors Experienced by Emergency Department Patients: Differences by Sexual Orientation and Gender Identification. J Interpers Violence. 2021;36(11-12):NP6125-NP6143. doi:10.1177/0886260518812070
International Association of Chiefs of Police. Successful Trauma Informed Victim Interviewing. Updated June 5, 2020. Accessed October 29, 2023. https://www.theiacp.org/resources/document/successful-trauma-informed-victim-interviewing.
Fast Facts: What is Intimate Partner Violence? Centers for Disease Control and Prevention. Updated October 11, 2022. Accessed August 30, 2022. https://www.cdc.gov/violenceprevention/intimatepartnerviolence/fastfact.html.
ACOG Committee Opinion No. 518: Intimate partner violence. Obstet Gynecol. 2012;119(2 Pt 1):412-417. doi:10.1097/AOG.0b013e318249ff74
Khurana B, Prakash J, Loder RT. Assault related injury visits in US emergency departments: An analysis by weekday, month and weekday-by-month. Chronobiol Int. 2022;39(8):1068-1077. doi:10.1080/07420528.2022.2065285
Kwan J, Sparrow K, Facer-Irwin E, Thandi G, Fear NT, MacManus D. Prevalence of intimate partner violence perpetration among military populations: A systematic review and meta-analysis. Aggress Violent Behav. 2020;53:101419. doi:10.1016/j.avb.2020.101419
Menschner C, Maul A. Key Ingredients for Successful Trauma-Informed Care Implementation. Center for Healthcare Strategies; Published April 2016. Accessed August 30, 2022. https://www.samhsa.gov/sites/default/files/programs_campaigns/childrens_mental_health/atc-whitepaper-040616.pdf.
National Domestic Violence Hotline. Accessed August 30, 2022. https://www.thehotline.org/.
National Resource Center on Domestic Violence. Resources. NRCDV. Updated 2021. Accessed August 30, 2022. https://www.nrcdv.org/resources.
Office of Violence Against Women. Sexual Assault. U.S. Department of Justice. Updated May 8, 2023. Accessed August 30, 2022. https://www.justice.gov/ovw/sexual-assault#:~:text=The%20term%20%E2%80%9Csexual%20assault%E2%80%9D%20means,victim%20lacks%20capacity%20to%20consent.
SAMHSA’s Trauma and Justice Strategic Initiative. SAMHSA’s Concept of Trauma and Guidance for a Trauma-Informed Approach. Published 2014. Accessed August 30, 2022. https://store.samhsa.gov/product/SAMHSA-s-Concept-of-Trauma-and-Guidance-for-a-Trauma-Informed-Approach/SMA14-4884.
Sexual Harassment Assault Response and Prevention (SHARP) - Benefit Fact Sheet. The Official Army Benefits Website. Published 2021. Updated September 29, 2022. Accessed August 30, 2022. https://myarmybenefits.us.army.mil/Benefit-Library/Federal-Benefits/Sexual-Harassment-Assault-Response-and-Prevention-(SHARP)?serv=122.
Trauma-Informed Care. Agency for Healthcare Research and Quality. Updated April 2016. Accessed August 30, 2022. https://archive.ahrq.gov/ncepcr/tools/healthier-pregnancy/fact-s
